# CXCL13 promotes broad immune responses induced by circular RNA vaccines

**DOI:** 10.1073/pnas.2406434121

**Published:** 2024-10-22

**Authors:** Jiawu Wan, Caiqian Wang, Zongmei Wang, Lingli Wang, Haoran Wang, Ming Zhou, Zhen F. Fu, Ling Zhao

**Affiliations:** ^a^National Key Laboratory of Agricultural Microbiology, Huazhong Agricultural University, Wuhan 430070, China; ^b^Hubei Hongshan Laboratory, Wuhan 430070, China; ^c^Frontiers Science Center for Animal Breeding and Sustainable Production, Wuhan 430070, China; ^d^Key Laboratory of Preventive Veterinary Medicine of Hubei Province, College of Veterinary Medicine, Huazhong Agricultural University, Wuhan 430070, China

**Keywords:** broadly cross-reactive antibodies, CXCL13, circRNA vaccine, influenza virus, SARS-CoV-2

## Abstract

Antibody responses induced by current vaccines for influenza and SARS-CoV-2 often lack robust cross-reactivity. As hubs where diverse immune cells converge and interact, the alterations in the immune microenvironment within lymph nodes (LNs) are intricately linked to immune responses. Herein, we designed a lipid nanoparticle (LNP) loaded with circular RNA (circRNA) and targeted to LNs, in which CXCL13 was directly integrated into antigen-encoding circRNA strands. We demonstrated that CXCL13 alters the transcriptomic profiles of LNs, especially the upregulation of IL-21 and IL-4. Meanwhile, CXCL13 promotes the formation of germinal center and elicits robust antigen-specific T cell responses. With the codelivery of CXCL13 and the antigen, CXCL13 enhances cross-reactive antibodies against influenza virus and SARS-CoV-2, achieving protection against both homologous and heterologous influenza virus challenges in a mouse model. Notably, the targeted modification of LNP surfaces with antibodies helps address some of the challenges associated with lyophilized LNP vaccines, which is crucial for the long-term storage of LNP-circRNA vaccines. Overall, the circRNA-based antigen-CXCL13 coexpression system developed herein provides a simple and robust platform that enhances the magnitude and breadth of antibody responses against multiple viral glycoproteins, highlighting the potential utility of CXCL13 in inducing broad immune responses.

## Significance

Integrating adjuvants into circular RNAs (circRNAs) demonstrates the prospect of developing circRNA vaccines and adjuvants, enabling circRNA encoding antigens and adjuvants can codeliver both components to the same antigen-presenting cell in a straightforward formulation. Here, we designed a general strategy to improve the magnitude and breadth of the antibody response induced by circRNA vaccines and remodeled the immune microenvironment of lymph nodes by integrating CXCL13 and antigens into circRNAs for coexpression. The targeted modification of the lipid nanoparticle (LNP) surface with antibodies effectively improves the lyophilization stability, enabling long-term preservation of the vaccine. This study demonstrates that CXCL13 based on the circRNA-tLNPs can provide broad immune stimulation in vaccines against multiple pathogens.

Messenger RNA (mRNA) vaccines are a key technology in combating existing and emerging infectious diseases (1, 2). Major advances have been made over the past 3 y in the application of mRNA vaccines to the development of vaccines, and circular RNA (circRNA) vaccines with higher stability have also been widely studied (3–5). However, vaccine development still faces many challenges, including the effectiveness, durability, and breadth of immune responses. The current highly active mutations of the influenza virus have resulted in a limited breadth and effectiveness of current seasonal influenza vaccines, making it challenging to develop effective preventive influenza vaccines (6). Similar to the influenza virus, the numerous variants of severe acute respiratory syndrome coronavirus 2 (SARS-CoV-2) have been the main reason for the ongoing COVID-19 pandemic for the past 3 y (7–9). As such, it is critical to develop vaccines with broadly protective capabilities to induce cross-reactive antibody responses against multiple viral variants.

Many studies have shown the advantages of combining the antigen and immunostimulatory adjuvant into a single formulation, guaranteeing the simultaneous delivery of these two key components to the same antigen-presenting cells (10, 11). Integrating adjuvants and antigens within the same circRNA strands instead of using separate adjuvant molecules is advantageous for formulating clinically translatable vaccines due to its simplified production process and lower cost advantages (12). This strategy is undoubtedly simpler and more efficient than redeveloping delivery materials and adjuvant molecules, but adjuvant molecules that can be coexpressed with antigens in circRNA are the premise. CXCL13 [chemokine (C-X-C motif) ligand 13] is capable of chemotaxis of B lymphocytes and Tfh cells by interacting with its receptor CXCR5, promoting the formation of germinal center (GC) and thereby coordinating humoral immunity (13, 14). Our previous work has demonstrated that CXCL13 can recruit Tfh cells and GC B cells, promoting the formation of GCs, thereby enhancing the intensity of immune responses (15). Meanwhile, previous research has found that plasma CXCL13 is associated with the production of HIV broadly neutralizing antibodies (bnAbs), with elevated levels of CXCL13 observed in the body following immunization (16). After influenza virus infection, type I interferon generated can induce the expression of CXCL13 in the lungs, initiating the formation of ectopic germinal centers, which in turn produce unique broad and protective neutralizing antibody responses (17). Nasal immunization with CTA1-3M2e-DD in adult mice stimulated robust heterosubtypic protection against influenza infection, while CTA1-DD greatly up-regulated the expression of the *Cxcl13* gene (18). These findings underscore the potential role of CXCL13 in inducing the production of bnAbs.

Compared to the significant advancements in the efficacy of mRNA vaccines, research on the stability and preservation of mRNA vaccines has lagged behind (19, 20). Even with more stable circRNAs, the storage duration remains relatively short. Currently authorized vaccines such as BNT162b2 (−80 °C to −60 °C) and mRNA-1273 (−20 °C) require low-temperature storage and transportation (21). Currently, the long-term storage of lyophilized LNP-mRNA vaccines at room temperature has been achieved by an increasing number of laboratories, and lyophilization represents a promising preservation method (19, 22–25). However, the mechanical stresses introduced during the lyophilization and rehydration processes may result in lipid membrane damage, leading to mRNA leakage (22). While lyophilization protective agents such as sucrose can be added, the damage caused by lyophilization still persists, posing a potential threat to the stability of mRNA-LNP vaccine efficacy. Meanwhile, the targeting modifications of current mRNA-LNP vaccines are increasingly recognized for their role in enhancing immune responses (26). For vaccines that have been surface-modified with targeting ligands on LNPs, the impact of lyophilization on the targeting stability of the vaccine remains to be studied, as the disruption of targeting modifications directly affects the efficacy of the vaccine.

In this study, we introduced an approach involving the coexpression of the self-adjuvanted CXCL13 circRNA. This research identified CXCL13 as an effective immunostimulatory component for both circRNA vaccines and protein subunit vaccines. The CXCL13 and antigen coexpression system induced robust cross-reactive antibody responses against multiple heterologous viral variants. Additionally, surface-modified antibodies on LNPs not only facilitated effective lymph nodes (LNs) targeting of the nanoparticle vaccines but also enhanced their lyophilization stability, extending storage time. Due to the intrinsic adjuvant properties of CXCL13, antigen-CXCL13-circRNA may offer a higher level of safety, as there is no need to add exogenous adjuvants. These findings highlight the potential of CXCL13 as a vaccine adjuvant for inducing broad immune protection. Additionally, one advantage of this antigen-adjuvant-circRNA vaccine is the flexibility to encode different adjuvant molecules through RNA, making it adaptable for various applications.

## Results

### LN Delivery of circRNA Encoding CXCL13 and HA Enhances Immune Responses.

**Fig. 1.**
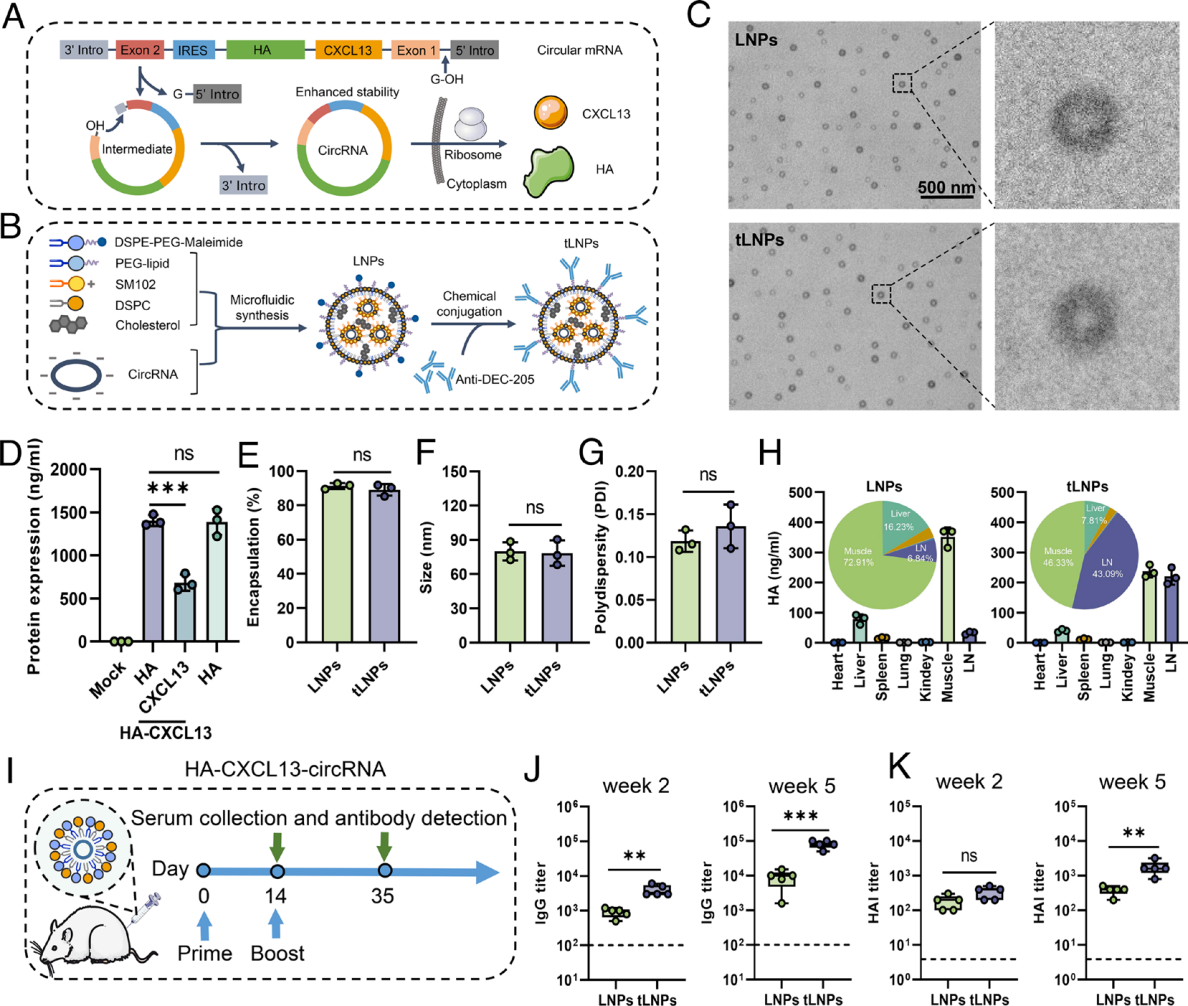
Design and characterization of HA-CXCL13-circRNA tLNPs vaccines targeted to LNs. (*A*) Schematic diagram of circRNA circularization by group I intron autocatalysis. When circRNA enters the cell, the internal ribosome entry site (IRES) sequence recognizes the ribosome, facilitating the direct completion of the protein expression process. (*B*) Schematic diagram of the construction of LN targeted-LNPs (tLNPs). The targeting antibodies (anti-DEC-205) are chemically conjugated with the LNPs to the maleimide group in the lipid DSPE-PEG-maleimide. (*C*) Representative transmission electron microscopy image of LNPs or tLNPs. (Scale bar, 500 nm.) (*D*) CircRNA was transfected into HEK-293T cells, and the protein concentration in HEK-293T cells was measured by ELISA 48 h after transfection. (*E*–*G*) Encapsulation efficiency (*E*), particle size (*F*), and polydispersity index (PDI) (*G*) of LNPs and tLNPs. (*H*) The antigen distribution of HA-circRNA LNPs and HA-circRNA tLNPs in various tissues. The pie chart represents the average values from three independent experiments. Each segment of the pie chart reflects the proportionate data calculated based on these replicates. (*I*-*K*) Schematic illustration of the experimental design (*I*). BALB/c mice (n = 5) were immunized twice with 10 µg of HA-CXCL13-circRNA LNPs or HA-CXCL13-circRNA tLNPs. Serum samples were collected at 14 and 35 d after the primary immunization for IgG titer (*J*) and hemagglutination inhibition (HAI) titer (*K*) detection. Data are represented as the mean ± SD. Unpaired two-tailed Student’s *t* test was performed for comparison, as indicated in the figures; **P < 0.01; ***P < 0.001; ns, not significant.

Given the potential of CXCL13 to induce bnAbs, we explored the feasibility of integrating CXCL13 into circRNA chains. As previously described (27–29), we integrated viral antigen and CXCL13 into the same circRNA for coexpression based on an LNP-encapsulated circRNA platform (Fig. 1*A*). DEC-205 is primarily expressed on dendritic cells, and several studies have utilized anti-DEC-205 antibody for targeted delivery of antigens to dendritic cells in vivo (30, 31). For the purpose of facilitating the action of CXCL13 within draining LN (dLN), the anti-DEC-205 antibody was chemically conjugated to the LNPs surface using maleimide/thiol chemistry, here termed targeted-LNPs (tLNPs) (Fig. 1*B*). Transmission electron microscopy imaging showed that both LNPs were round-shaped nanoparticles (Fig. 1*C*). Transfection of HEK-293T cells revealed that both HA-CXCL13-circRNA and HA-circRNA generated similar amounts of influenza virus hemagglutinin (HA) protein (Fig. 1*D*). Furthermore, to verify whether tLNP-encapsulated HA-CXCL13-circRNA enables the expression of HA and CXCL13 proteins in the same cells (especially within dendritic cells), we collected inguinal LNs from mice immunized with HA-CXCL13-circRNA tLNPs at different time points and performed multiplex immunofluorescence staining. As shown in the results, HA and CXCL13 proteins were abundantly colocalized with CD11c-positive dendritic cells after intramuscular injection (*SI Appendix*, Fig. S1). In addition, most of HA and CXCL13 proteins were located in the medullary region and also but to a lesser extent in the interfollicular region or subcapsular sinus (*SI Appendix*, Fig. S2). These findings indicate that HA-CXCL13-circRNA tLNP formulations can effectively deliver HA and CXCL13 components to the same antigen-presenting cells.

LNPs and tLNPs exhibited similar encapsulation efficiency (Fig. 1*E*), particle size (Fig. 1*F*), and PDI (Fig. 1*G*), indicating that modification with anti-DEC-205 antibodies does not affect the self-assembly of LNPs. Simultaneously, this targeted modification altered the biodistribution of antigen expression in vivo. HA-CXCL13-circRNA LNPs exhibited a HA expression of only 6.84% in the dLNs, whereas HA-CXCL13-circRNA tLNPs increased to 43.09%, an approximately 6.3-fold increase (Fig. 1*H*). Notably, after modification of the LNPs by targeting dLNs, the proportion of antigen expression in the liver decreased from 16.23% to 7.81% (Fig. 1*H*). Therefore, the targeted delivery of circRNA in vivo can minimize vaccine accumulation in the liver. Further in vivo validation was conducted to verify whether this targeted modification has a promotional effect on the immune response (Fig. 1*I*). The data indicate that the HA-CXCL13-circRNA tLNPs vaccine elicits significantly higher antibody levels compared to the HA-CXCL13-circRNA LNPs vaccine (Fig. 1 *J* and *K*).

### CXCL13 Induced the Reprogramming of LN Transcriptome.

**Fig. 2.**
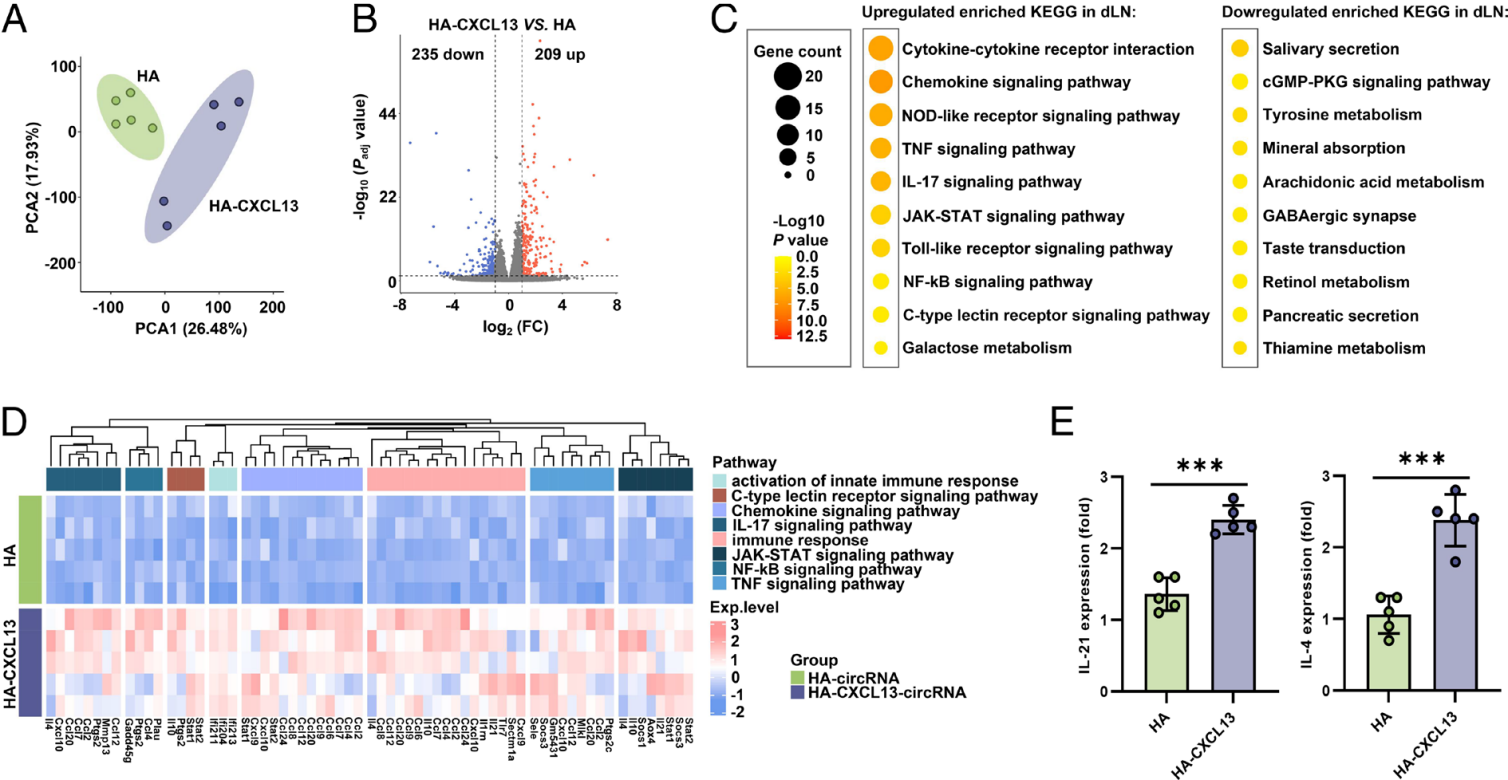
Transcriptome analysis of LNs revealed distinct immune response between CXCL13 expression and control mice. C57BL/6 mice (n = 5) were immunized via intramuscular injection with 10 µg of HA-CXCL13-circRNA tLNPs or HA-circRNA tLNPs. Two weeks after the initial immunization, a booster dose of the same amount was administered. Inguinal dLNs were collected 2 d after the booster immunization for RNA sequencing. (*A*) The PCA model plot of transcriptomic profiles from the inguinal dLNs of mice inoculated with HA-CXCL13-circRNA tLNPs and HA-circRNA tLNPs. PCA Plot showing the global differences between CXCL13 expression and control groups. (*B*) Volcano plots of differentially expressed genes between different groups. The red dots represent genes that are up-regulated, the blue dots represent genes that are down-regulated, and the gray dots represent genes that are not different. FDR < 0.01, |log_2_ (fold change)| > 1. (*C*) KEGG enrichment analysis of differentially expressed genes. The data are represented as circles, where the size indicates the gene count for that particular process, and the color represents the –Log_10_ *P*-value calculated with one-sided Fisher’s exact test with Benjamini–Hochberg correction. (*D*) Heatmap visualization of scaled gene expression levels for selected pathways of interest. (*E*) The expression of IL-21 and IL-4 in mouse LNs inoculated with HA-circRNA tLNPs or HA-CXCL13-circRNA tLNPs. Data are represented as the mean ± SD. Unpaired two-tailed Student’s *t* test was performed for comparison, as indicated in the figures; ***P < 0.001. PCA: principal component analysis; KEGG: Kyoto Encyclopedia of Genes and Genomes; FDR: false discovery rate.

The primary role of CXCL13 in the GC is to attract activated antigen-specific B cells and Tfh cells to the B cell follicles and further into GC (32, 33). Based on our above results of CXCL13-circRNA tLNPs targeting dLNs, we hypothesized that exogenous CXCL13 may interact with various immune cells in the dLNs. To investigate whether the coexpression of CXCL13 with antigens affects the microenvironment of the dLNs, we examined the changes in the transcriptome of dLNs in vaccinated mice. The principal component analysis (PCA) plot demonstrates significant differences between the CXCL13 treatment group and the control group (Fig. 2*A*), with 209 genes up-regulated and 235 genes down-regulated (Fig. 2*B*), indicating substantial changes in the transcriptome of mouse dLNs after CXCL13 inoculation. After CXCL13 inoculation, there is extensive enrichment in adaptive immunity, chemokine activity, cytokine activity, NOD-like receptor signaling pathway, and inflammatory responses (TNF signaling pathway, IL-17 signaling pathway, JAK-STAT signaling pathway, NF-kB signaling pathway) (Fig. 2 *C* and *D* and *SI Appendix*, Fig. S3). Chemokines serve as crucial cues directing immune cell localization in both homeostasis and inflammatory responses, with the chemokine microenvironment supporting the compartmentalization of LN tissues into distinct zones (34). Antiviral innate immune pathways such as Toll-like receptor signaling pathway are significantly activated. Further data confirmed that compared to mice inoculated with HA-circRNA, the expression of IL-21 and IL-4 increased in mice inoculated with HA-CXCL13-circRNA (Fig. 2*E*). These results indicate that CXCL13 inoculation alters the overall transcriptional profile of mouse dLNs.

### CXCL13 Induces Robust and High-Level Germinal Center Responses.

**Fig. 3.**
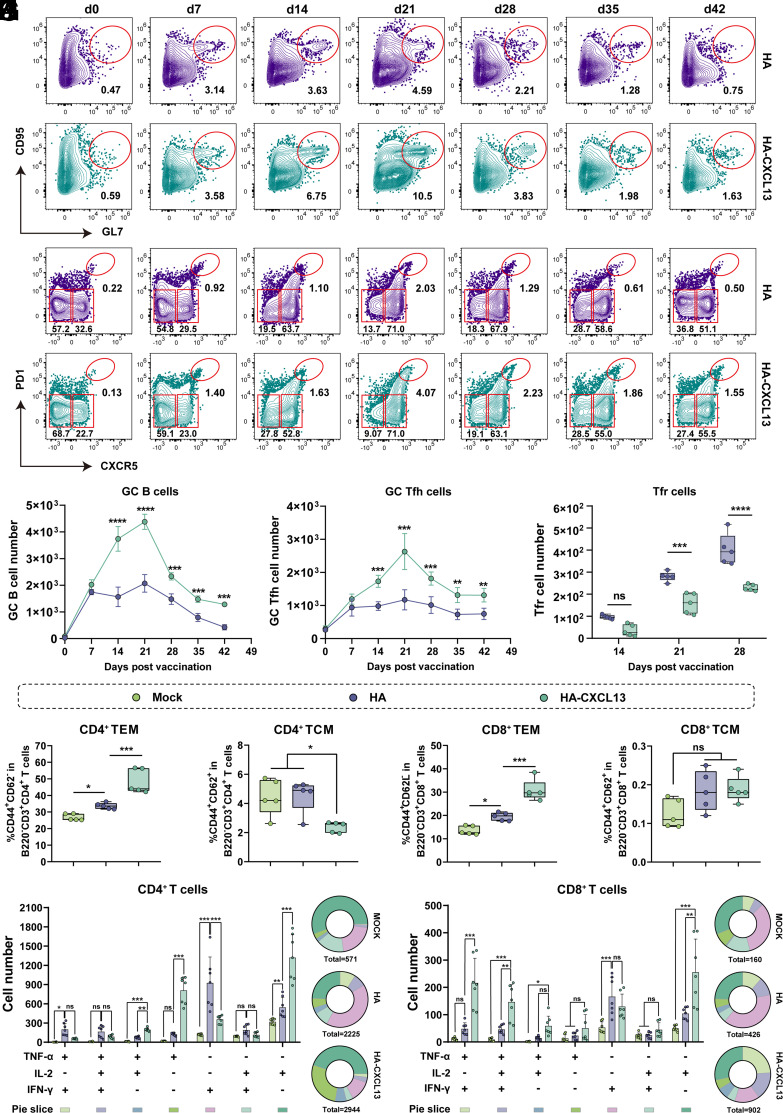
HA-CXCL13-circRNA enhanced both humoral and cellular immune responses. C57BL/6 mice were immunized with HA-CXCL13-circRNA tLNPs or HA-circRNA tLNPs on days 0 and 14, and inguinal dLNs were harvested for FCM analysis. (*A*) Representative flow cytometry plots of GC B cells (gated on CD3^−^B220^+^CD95^+^GL7^+^ cells) in dLNs from days 0, 7, 14, 21, 28, 35, and 42. See *SI Appendix*, Fig. S4 for the full gating strategy. (*B*) Representative flow cytometry plots of GC Tfh cells (gated on B220^−^CD3^+^CD8^−^CD4^+^CXCR5^hi^PD-1^hi^ cells), mantle Tfh (mTfh, gated on B220^−^CD3^+^CD8^−^CD4^+^CXCR5^int^PD-1^int^), and non-Tfh (gated on B220^−^CD3^+^CD8^−^CD4^+^CXCR5^−^) in dLNs from days 0, 7, 14, 21, 28, 35, and 42. See *SI Appendix*, Fig. S4 for the full gating strategy. (*C*) Numbers of GC B cells over time (n = 5 mice per time point). (*D*) Numbers of GC Tfh cells over time (n = 5 mice per time point). (*E*) Total number of Tfr cells (gated on B220^−^CD3^+^CD8^−^CD4^+^PD-1^+^BCL6^hi^FoxP3^+^) in dLNs from days 14, 21, and 28 (n = 5 mice per time point). (*F* and *G*) Statistical results of CD4^+^ TEM (gated on B220^−^CD3^+^CD8^−^CD4^+^CD44^+^CD62L^−^) and TCM (gated on B220^−^CD3^+^CD8^−^CD4^+^CD44^+^CD62L^+^) in the spleen at day 21 (n = 5 mice per group). (*H* and *I*) Statistical results of CD8^+^ TEM (gated on B220^−^CD3^+^CD4^−^CD8^+^CD44^+^CD62L^−^) and TCM (gated on B220^−^CD3^+^CD4^−^CD8^+^CD44^+^CD62L^+^) in the spleen at day 21 (n = 5 mice per group). (*J* and *K*) Number of cytokine-producing CD4^+^ T cells (*J*) and CD8^+^ T cells (*K*) in the spleen at day 21 postimmunization (n = 7 mice per group). Pie charts representing the proportions of cytokine-producing cells positive for one, two, or three cytokines. See *SI Appendix*, Fig.S5 for the full gating strategy. Data are represented as the mean ± SD. Statistical significance was determined by one-way or two-way ANOVA with Tukey’s multiple comparisons; *P < 0.05; **P < 0.01; ***P < 0.001; ****P < 0.0001; ns, not significant.

The GC response is a critical process that occurs during dLNs after vaccine immunization, the core of which is the interaction between GC B cells and germinal center T follicular helper cells (GC-Tfhs) (35, 36). Previous research has shown that CXCL13 signals could drive the development of secondary lymphoid tissue and the trafficking of B cells and Tfh cells to GC via the G protein-coupled chemokine receptor CXCR5 (32). To explore whether the addition of CXCL13 to the vaccine affects the GC response, we immunized mice with HA-CXCL13-circRNA tLNPs or HA-circRNA tLNPs on days 0 and 14, and monitored GC dynamics in the inguinal dLN for six consecutive weeks after the primary immunization. As shown in Fig. 3 *A* and *C*, both vaccines produced a similar number of GC B (CD3^−^B220^+^CD95^+^GL7^+^) cells on day 7, but the number of GC B cells in the HA-circRNA group started to decline on day 14, continued to expand after the booster and peaking at day 21, subsequently contracted between days 28 and 42. It is noteworthy that the HA-CXCL13-circRNA group did not show a decline on day 14, but continued to expand. Importantly, high-frequency GC B cells were still detectable in mice immunized with HA-CXCL13-circRNA on day 42, and the number was about three times that of the HA-circRNA group. Moreover, mice immunized with HA-CXCL13-circRNA instead of HA-circRNA generated high levels of HA-specific GC B (CD3^−^B220^+^CD95^+^GL7^+^HA^+^) cells and switched MBCs (CD3^−^B220^+^IgD^-^CD38^+^HA^+^) at day 21 after primary immunization (*SI Appendix*, Fig. S6 *A–D*).

Consistent with GC B cell dynamics, GC Tfh (CD8^−^CD4^+^ CXCR5^hi^PD1^hi^) cells were also highly expanded in HA-CXCL13-circRNA immunized mice and peaked on day 21 (Fig. 3 *B* and *D*). Previous studies have shown that antigen-experienced CD4^+^ T cells in lymphoid tissues are divided into GC Tfh (CD4^+^CXCR5^hi^PD-1^hi^), follicular mantle Tfh (mTfh, CD4^+^CXCR5^int^PD-1^int^), and non-Tfh (CD4^+^CXCR5^-^) (16, 37). When the CD4^+^ T cells in inguinal LNs were characterized into these three subsets, we observed that their total frequencies were in a dynamic equilibrium state, fluctuating around 48% (*SI Appendix*, Fig. S6 *E* and *F*). Interestingly, the kinetics pattern of GC Tfh was similar to that of mTfh, but opposite to that of non-Tfh (*SI Appendix*, Fig. S6 *H* and *I*).

GC responses need to be highly regulated to prevent the production of autoantibodies and the development of systemic autoimmune diseases (38). Recent studies have shown that the regulation of Tfh and B cell responses mediated by follicular regulatory T (Tfr) cells is critical for appropriate GC responses (39). We further examined the number of Tfr (CD8^−^CD4^+^BCL6^+^PD1^hi^FoxP3^+^) cells before and after booster immunization. We found that Tfr cells were in a continuous growth trend, but the growth rate in the HA-CXCL13-circRNA group was comparatively slower (Fig. 3*E*). Additionally, the ratio of Tfr cells and Tfh cells at the corresponding time points was lower in HA-CXCL13-circRNA group (*SI Appendix*, Fig. S6*G*). Given that Tfr cell subsets have been shown to migrate to GC and specifically and effectively inhibit Tfh and GC B cells (39–41), we speculated that a low proportion of Tfr cells might be responsible for the maintenance of GCs in the HA-CXCL13-circRNA group. Taken together, these data suggest that CXCL13 can effectively promote GC reactions and prolong GC reactions to some extent.

### CXCL13 Promotes CD4^+^ and CD8^+^ T Cells Responses.

To assess the impact of adding CXCL13 on the cellular immune response, we immunized three groups of mice with HA-circRNA tLNPs, HA-CXCL13-circRNA tLNPs, and a placebo following the same immunization schedule as mentioned above. One week after the booster, the spleens were isolated and stimulated with HA protein in vitro. The production of IFN-γ, TNF-α, and IL-2 was detected by intracellular cytokine staining (ICCS), and HA-specific responding cells were characterized as antigen-experienced (CD44^+^) T cells expressing IFN-γ, TNF-α, and/or IL-2 (see *SI Appendix*, Fig. S5 for full gating strategy). We observed different CD4^+^ T cell response patterns induced by the two vaccines (Fig. 3*J*). Specifically, HA-CXCL13-circRNA induced more CD4^+^ T cells producing TNF-α (TNF-α^+^IL-2^−^IFN-γ^−^) or IL-2 (TNF-α^−^IL-2^+^IFN-γ^−^), while HA-circRNA generated more IFN-γ single positive cells. In contrast, mice immunized with HA-CXCL13-circRNA produced a greater number of TNF-α^+^IL-2^+^IFN-γ^−^ CD4^+^ T cells. The proportion of TNF-α^+^IFN-γ^+^ double positive and TNF-α^+^IL-2^+^IFN-γ^+^ triple positive CD4^+^ T cells was higher in the HA-circRNA group, although the differences were not significant. In general, HA-CXCL13-circRNA immunization elicited more CD4^+^ T cells expressing TNF-α and/or IL-2, but the proportion of IFN-γ^+^ CD4^+^ T cells was lower than that of HA-circRNA.

In addition, the CD8^+^ T cell responses in the different immunization groups were further analyzed. As shown in Fig. 3*K*, the IFN-γ single positive cells continued to be the majority population in the HA-circRNA group. However, unlike CD4^+^ T cells, TNF-α^+^IL-2^−^IFN-γ^−^ and TNF-α^+^IL-2^−^IFN-γ^+^ CD8^+^ T cell populations were significantly lower in mice vaccinated with HA-circRNA. Conversely, TNF-α^+^IFN-γ^+^ double positive and TNF-α^+^IL-2^+^IFN-γ^+^ triple positive cells were more prevalent among HA-specific CD8^+^ T cells elicited by HA-CXCL13-circRNA. It should be noted that the total number of multifunctional CD8^+^ T cells was larger in mice immunized with HA-CXCL13-circRNA, approximately twice as many as the HA-circRNA group. Furthermore, we found that HA-CXCL13-circRNA induced higher frequencies of CD4^+^ and CD8^+^ effector memory T (TEM, B220^−^CD3^+^CD4^+^/CD8^+^CD62L^−^CD44^+^) (Fig. 3 *F* and *H*), but CD4^+^ central memory T (TCM, B220^−^CD3^+^CD4^+^CD62L^+^CD44^+^) cells were significantly lower than those in the HA-circRNA group (Fig. 3*G*). Strangely, the frequency of CD8^+^ TCM detected in both immune groups is very low and there is no significant difference (Fig. 3*I*). Overall, our results indicate that HA-CXCL13-circRNA induced more CD4^+^ TEMs and enhanced the recruitment of TNF-α^+^, IL-2^+^, and TNF-α^+^IL-2^+^ CD4^+^ T cells to the spleen, as well as eliciting potent CD8^+^ T cell responses.

### CXCL13 Enhances Cross-Reactive and Stalk-Specific Antibody Responses.

**Fig. 4.**
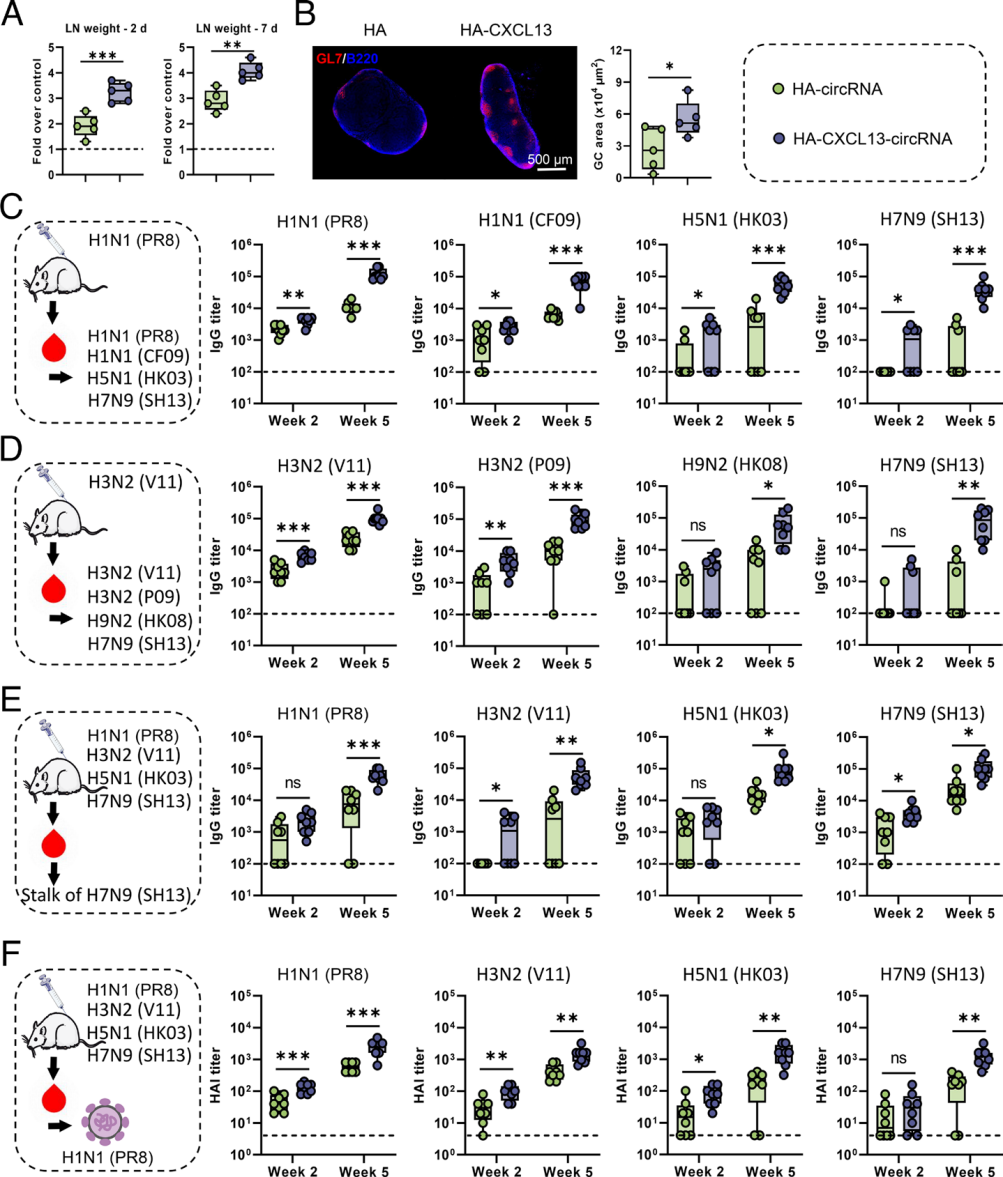
CXCL13-circRNA-adjuvanted HA-circRNA immunization elicits cross-reactive antibody responses. (*A*) BALB/c mice (n = 5) were intramuscularly injected with PBS, HA-CXCL13-circRNA tLNPs, or HA-circRNA tLNPs. After 2 or 7 d, the inguinal dLNs were collected and analyzed for weight. The results are presented as the fold of the LN weight from the PBS-injected contralateral side. (*B*) The GC response in the inguinal dLNs (day 10) was analyzed by immunofluorescence. LN sections were stained for B220 (blue) and GL7 (red). Sizes of GCs in representative immunofluorescent images (*Right* panel). (C–E) BALB/c mice (n = 8) were immunized by intramuscular injection with 10 µg of HA-CXCL13-circRNA tLNPs or HA-circRNA tLNPs and boosted with the same dose after 2 wk. Serum samples were collected at 14 and 35 d after primary immunization and measured by ELISA for antibody levels. (*C*) Serum IgG responses were measured against HA (PR8, CF09, HK03, and SH13) proteins after immunization with HA-CXCL13-circRNA or HA-circRNA (PR8) vaccines. (*D*) Serum IgG responses were measured against HA (V11, P09, HK08, and SH13) proteins after immunization with HA-CXCL13-circRNA or HA-circRNA (V11) vaccines. (*E*) Serum IgG responses were measured against stalk region of HA (SH13) proteins after immunization with HA-CXCL13-circRNA or HA-circRNA (PR8, V11, HK03, and SH13) vaccines. (*F*) BALB/c mice (n = 8) were immunized with HA-CXCL13-circRNA or HA-circRNA vaccines constructed from HA sequences of different influenza strains (PR8, V11, HK03, and SH13). After immunization, the HAI titers against H1N1 (PR8) in serum were measured. Data are represented as the mean ± SD. Unpaired two-tailed Student’s *t* test was performed for comparison, as indicated in the figures; *P < 0.05; **P < 0.01; ***P < 0.001; ns, not significant.

After evaluating the CXCL13-induced GC levels by flow cytometry, we further analyzed the impact of CXCL13 on the development of dLNs and GC formation. The results showed that compared to HA-circRNA, HA-CXCL13-circRNA promoted dLN development (Fig. 4*A*). Additionally, HA-CXCL13-circRNA induced more GCs on day 10 after immunization (Fig. 4*B*). We next immunized mice with HA-circRNA or HA-CXCL13-circRNA constructed from the influenza strain H1N1 A/Puerto Rico/8/1934 (PR8) HA sequence. Antibodies against HA from homologous and heterologous strains were examined at week 2 and week 5 postimmunization. The results demonstrated that HA-CXCL13-circRNA not only elevated antibody titers against HA from PR8 but also increased antibody titers against HA from another H1N1 strain, A/California/04/2009 (CF09) (Fig. 4*C*). Simultaneously, mice immunized with HA-CXCL13-circRNA generated antibodies against HA of the H5N1 A/Hong Kong/213/03 (HK03) and H7N9 A/Shanghai/02/2013 (SH13) strains, which are highly pathogenic influenza virus variants from two different subtypes. In contrast, these heterosubtypic antibodies were not detected in some mice (50 to 62.5%) immunized with HA-circRNA (Fig. 4*C*). Subsequently, we conducted similar experiments but immunized using circRNA constructed with HA from the H3N2 A/Victoria/361/2011 (V11) virus. We found that compared to HA-circRNA, HA-CXCL13-circRNA also induced significantly higher levels of antibodies against HA of cross-subtype or even cross-group (Fig. 4*D*). Importantly, in contrast to HA-circRNA, HA-CXCL13-circRNA can promote the generation of antibodies against the conserved but subdominant stalk region (Fig. 4*E*). With alterations in circRNA derived from HA of different strains, HA-CXCL13-circRNA still enhances the antibody response against the stalk region. To further examine the quality of antibody responses, we examined partial IgG subclasses. Both HA-circRNA and HA-CXCL13-circRNA induced high levels of IgG1 (*SI Appendix*, Fig. S7*A*), but mice immunized with HA-CXCL13-circRNA exhibited the strongest IgG2c production (*SI Appendix*, Fig. S7*B*).

To further evaluate the quality of vaccine-induced humoral immunity, we utilized the hemagglutination inhibition (HAI) assay to identify HA-specific functional antibody responses using the influenza virus PR8 (H1N1). Both the HA-circRNA and HA-CXCL13-circRNA vaccines, constructed with the PR8 strain sequence, induced high HAI titers, with HA-CXCL13-circRNA inducing significantly higher HAI titers than HA-circRNA (Fig. 4*F*). For the HA-circRNA vaccines constructed with the HA sequences from heterologous strains (H3N2, H5N1, H7N9), some mice had HAI titers below the detection threshold after the primary immunization and still failed to produce effective HAI titers even after booster immunization. For HA-CXCL13-circRNA vaccines constructed with HA sequences from heterologous strains, although some mice had HAI titers below the detection threshold after the initial immunization, effective and high HAI titers were induced following the booster immunization. Therefore, we demonstrated the potential of HA-CXCL13-circRNA in inducing cross-reactive antibody responses.

### CXCL13 Enhances Protection Against Homologous and Heterologous Influenza Infection.

**Fig. 5.**
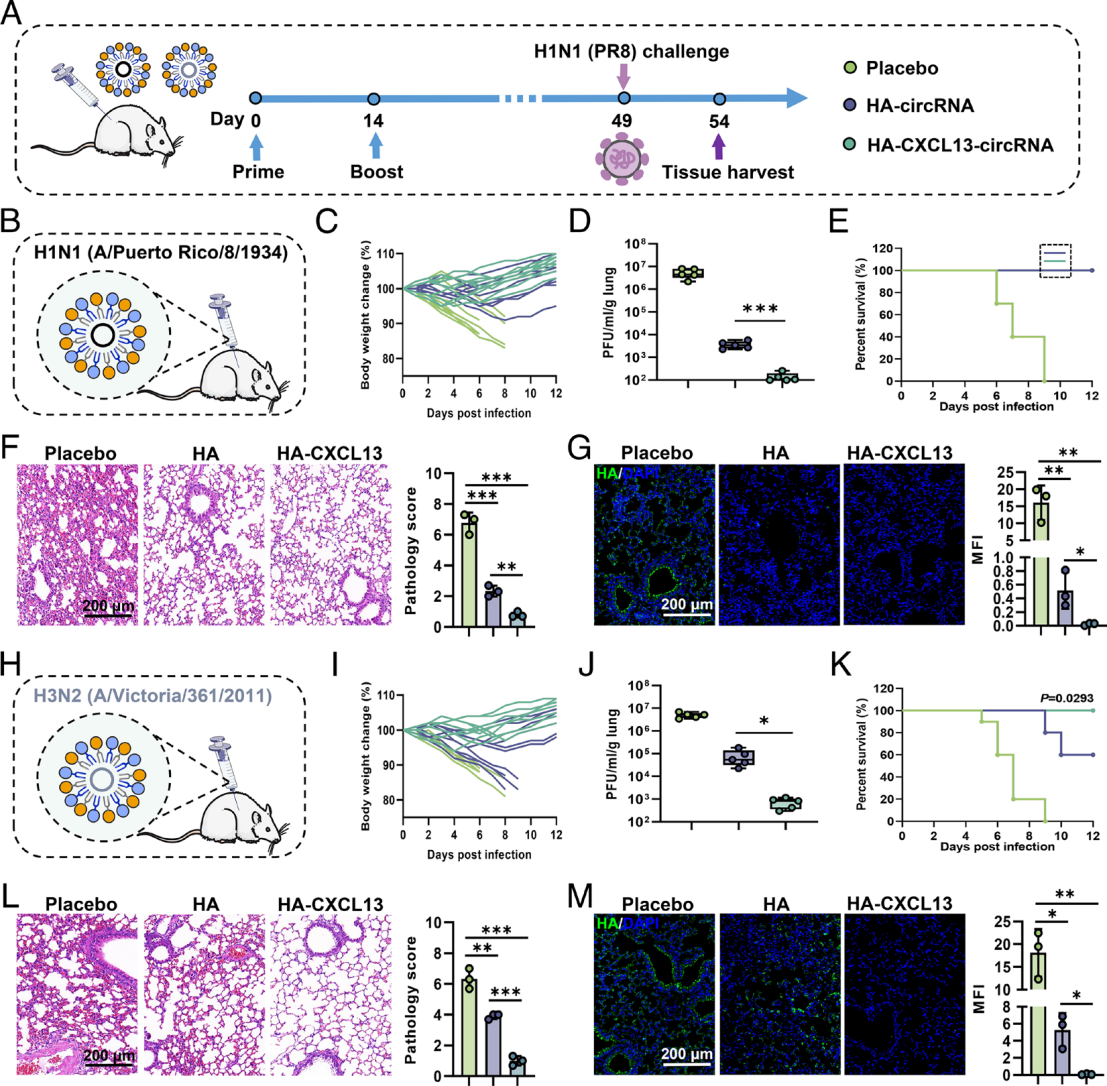
CXCL13 enhances protection against homologous and heterologous influenza virus challenge. (*A*) Schematic illustration of the experimental design. (*B*) Mice were immunized twice with HA-CXCL13-circRNA tLNPs or HA-circRNA tLNPs constructed using PR8 (H1N1). On day 49, mice were intranasally infected with PR8 (H1N1), and body weight changes (*C*), viral load (*D*), and survival (*E*) were recorded for 12 days. N = 10 (*C*, *E*) or 5 (*D*) mice per group. (*F*) Hematoxylin and eosin (H&E) staining and pathological scoring of the lung tissues from different groups of mice at 5 days postchallenge. (*G*) Immunostaining of lung tissues with an influenza virus HA-specific monoclonal antibody (mAb) and quantification of the mean fluorescence intensity (MFI). (*H*) Mice were immunized twice with HA-CXCL13-circRNA tLNPs or HA-circRNA tLNPs constructed using H3N2 (V11). Body weight changes (*I*), viral load (*J*), and survival (*K*) were recorded for 12 days after infection with PR8 (H1N1). N = 10 (*I*, *K*) or 5 (*J*) mice per group. (*L*) H&E staining and pathology scoring at 5 days postchallenge. (*M*) Immunostaining and MFI quantification. The data are presented as representative images from three independent visual fields randomly collected from each of the three mice per group. The pathology score for each mouse is the average score from three independent visual fields. The MFI for each mouse is the average value from three independent visual fields. Data are represented as the mean ± SD. Unpaired two-tailed Student’s *t* test was performed for comparison, as indicated in the figures; *P < 0.05; **P < 0.01; ***P < 0.001.

To explore the cross-protective efficacy against homologous and heterologous influenza viruses induced by CXCL13-circRNA, mice from the group immunized with two doses of PR8-derived HA-CXCL13-circRNA tLNPs or HA-circRNA tLNPs were intranasally challenged with PR8 (H1N1) virus at 7 wk after the primary immunization (Fig. 5*A*). During homologous virus challenge (Fig. 5*B*), there was no significant difference in protecting mice from weight loss between HA-CXCL13-circRNA and HA-circRNA, although slight weight loss was observed in the initial days after infection for HA-circRNA (Fig. 5*C*). Additionally, we found markedly reduced viral lung titers in mice immunized with HA-CXCL13-circRNA compared with placebo-treated or HA-circRNA-immunized mice (Fig. 5*D*). Furthermore, all mice that received two doses of either HA-CXCL13-circRNA or HA-circRNA immunization survived the influenza infection (Fig. 5*E*). Histopathological analyses demonstrated that mice vaccinated with HA-circRNA, although surviving the viral challenge, still observed very mild pneumonia induced by viral infection, whereas mice vaccinated with HA-CXCL13-circRNA avoided lung injury (Fig. 5*F*). In contrast, the placebo group mice showed severe interstitial pneumonia, as exemplified by thickened alveolar septa, alveolar congestion/edema, bronchial/bronchiolar inflammation, infiltration of inflammatory cells, and endothelial cell vacuolation. Immunofluorescence staining analyses further revealed minimal expression of HA protein in lung sections of mice immunized with HA-circRNA, whereas no viral protein expression was detected in mice immunized with HA-CXCL13-circRNA (Fig. 5*G*). The pathology scores and mean fluorescence intensity (MFI) further confirm that HA-CXCL13-circRNA and HA-circRNA can both protect mice from influenza virus infection. However, similar to the induced homologous antibody responses, HA-CXCL13-circRNA demonstrates superior protection against influenza virus infection in mice.

Next, we further evaluated the cross-protective ability of HA-CXCL13-circRNA against heterologous strains. In this case, mice were immunized with HA-CXCL13-circRNA tLNPs and HA-circRNA tLNPs based on the HA sequence from the H3N2 strain and then challenged with PR8 (H1N1) virus (Fig. 5*H*). Mice immunized with HA-CXCL13-circRNA exhibited less weight loss and lower viral loads when challenged with heterologous virus than mice immunized with HA-circRNA (Fig. 5 *I* and *J*). Mice immunized with HA-circRNA failed to generate effective cross-protection against the PR8 (H1N1) virus, as only 60% of the mice survived the challenge (Fig. 5*K*). In contrast, mice immunized with HA-CXCL13-circRNA remained completely alive and free of disease symptoms upon exposure to the heterologous virus challenge. Immunofluorescence staining and histopathological analyses revealed extensive viral infection and tissue damage in the lungs of mice immunized with HA-circRNA (Fig. 5 *L* and *M*). As expected, mice immunized with the HA-CXCL13-circRNA vaccine did not exhibit typical pathological changes after infection, and the virus was cleared postinfection. In conclusion, these findings demonstrate the superior ability of HA-CXCL13-circRNA in inducing cross-protection against both homologous and heterologous influenza viruses.

The superior ability of the two-dose HA-CXCL13-circRNA tLNPs vaccine in inducing cross-protection against homologous and heterologous influenza viruses has allowed us to further explore the protective efficacy of a single-dose HA-CXCL13-circRNA tLNPs vaccine. Following single-dose immunization, we conducted antibody detection and influenza virus challenge experiments. The results showed that compared to HA-circRNA, HA-CXCL13-circRNA induced higher levels of cross-reactive antibodies (*SI Appendix*, Fig. S8 *A* and *B*). However, it was evident that the antibody titers induced by a single dose of HA-CXCL13-circRNA were lower than those induced by the two-dose regimen. In subsequent influenza virus challenge tests, both the HA-circRNA and HA-CXCL13-circRNA groups of mice maintained a 100% survival rate against homologous influenza virus infection (*SI Appendix*, Fig. S8*C*). Notably, when challenged with heterologous influenza virus, 70% of the mice in the HA-circRNA group died, compared to a 40% mortality rate in the two-dose HA-circRNA vaccine group, indicating a 30% increase in mortality with the single-dose HA-circRNA. Similarly, in the single-dose HA-CXCL13-circRNA group, one mouse succumbed to heterologous influenza virus infection (*SI Appendix*, Fig. S8*D*). These results indicate that although the single-dose HA-CXCL13-circRNA provided complete protection against homologous influenza virus infection and 90% protection against heterologous influenza virus infection, there is still a low-risk infection when facing heterologous influenza virus compared to the two-dose regimen. In another highly lethal rabies virus model, we tested the immunogenicity of a single dose of G-CXCL13-circRNA based on the rabies virus glycoprotein (G). Although both G-circRNA and G-CXCL13-circRNA induced complete protection against rabies virus, CXCL13 significantly enhanced antibody levels (*SI Appendix*, Fig. S8 *E* and *F*), which is beneficial for further reducing vaccine dosage while maintaining complete protection against rabies virus (*SI Appendix*, Fig. S8*G*).

To further evaluate the safety of HA-CXCL13-circRNA tLNPs vaccines in mice, physiological and biochemical indicators were monitored. BALB/c mice were immunized via the intramuscular route with phosphate-buffered saline (PBS) or HA-CXCL13-circRNA tLNPs vaccines. Blood samples were collected for biochemical analysis on days 1, 7, and 14 postimmunization. Furthermore, major organs were collected for histopathological analysis 14 d postimmunization. H&E staining images revealed that, in comparison to the PBS group of mice, there were no significant histopathological changes observed in the major organs of mice immunized with the HA-CXCL13-circRNA tLNPs vaccines (*SI Appendix*, Fig. S9*A*). Furthermore, liver and kidney function markers, including aspartic acid aminotransferase (AST) and total protein (TP), in mice receiving the vaccine exhibited values that were elevated beyond the normal range at certain time points within the first and seventh days postimmunization, but subsequently returned to normal levels (*SI Appendix*, Fig. S9*B*). Additionally, hematological parameters in mice immunized with the vaccine were found to be similar to those of the PBS control group, with no significant differences observed (*SI Appendix*, Fig. S9*C*). Collectively, our study provides preliminary data of safety for the HA-CXCL13-circRNA tLNPs vaccination in mice.

### CXCL13 Enhances Both the Magnitude and Breadth of the Antibody Response Against SARS-CoV-2 Variants.

**Fig. 6.**
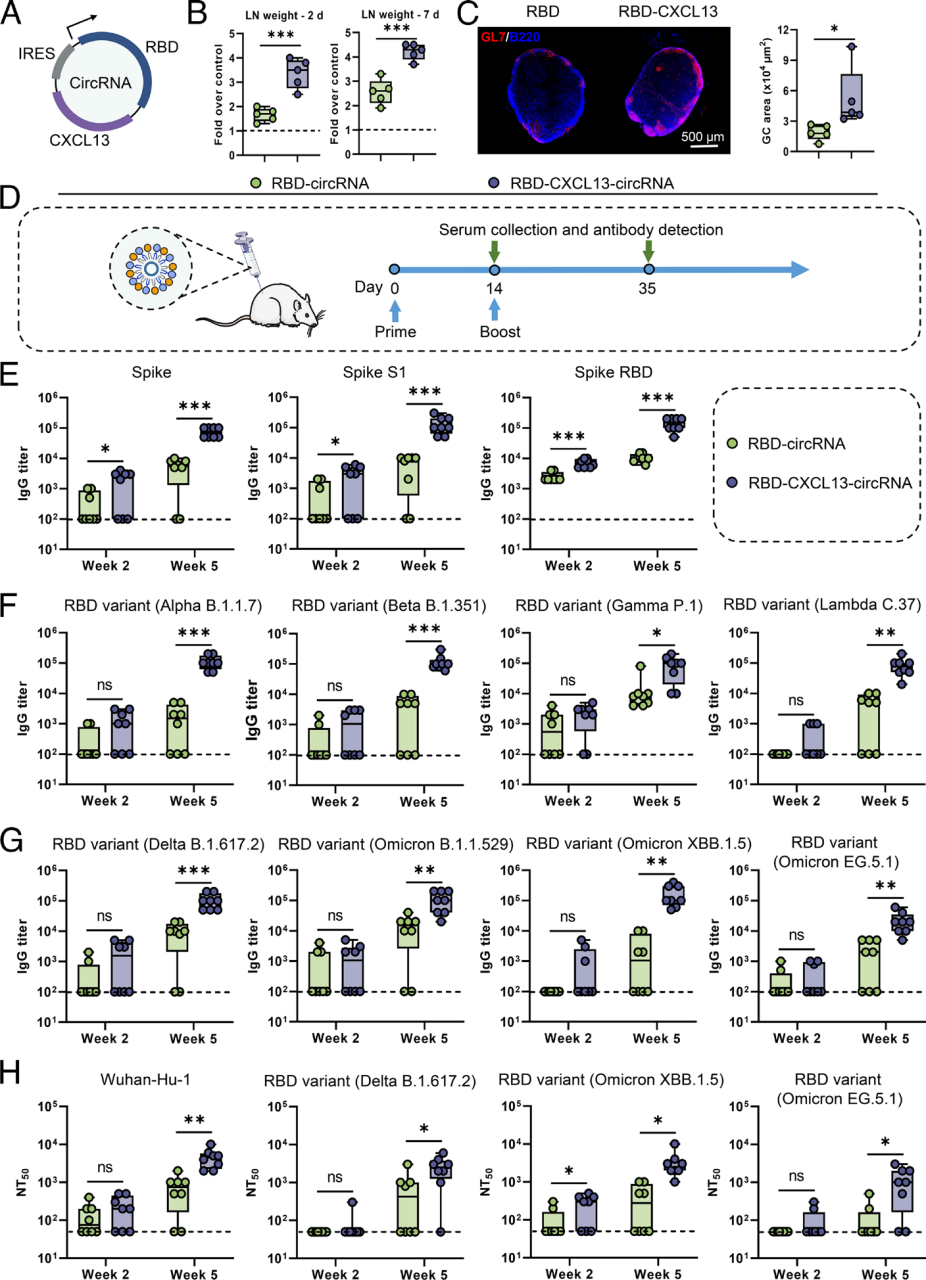
CXCL13-circRNA-adjuvanted SARS-CoV-2 RBD-circRNA immunization induces cross-reactive antibodies against multiple viral variants. (*A*) Schematic diagram of the circRNA, encoding the SARS-CoV-2 trimeric RBD and CXCL13. The arrow indicates the expected open-reading frame. IRES, internal ribosome entry site. (*B*) Mice were intramuscularly injected with PBS, RBD-CXCL13-circRNA tLNPs, or RBD-circRNA tLNPs. After 2 or 7 d, the inguinal dLNs were collected and analyzed for weight. The results are presented as the fold of the LN weight from the PBS-injected contralateral side. N = 5 mice per group. (*C*) The GC response in the inguinal dLNs (day 10) was analyzed by immunofluorescence. LN sections were stained for B220 (blue) and GL7 (red). Sizes of GCs in representative immunofluorescence images (*Right* panel). (*D*) Schematic illustration of the experimental design. Groups of BALB/c mice were immunized by intramuscular injection with 10 µg of RBD-CXCL13-circRNA tLNPs or RBD-circRNA tLNPs, both based on the Wuhan-Hu-1 RBD sequence, and boosted with the same dose after 2 wk. Serum samples were collected at 14 and 35 d after the prime immunization for antibody detection. (*E*–*G*) Antibodies binding to spike RBD, spike S1 and spike (Wuhan-Hu-1) (*E*), and RBD from relevant variants (*F* and *G*) were analyzed by ELISA. N = 8 mice per group. (*H*) Mice were immunized with RBD-CXCL13-circRNA tLNPs or RBD-circRNA tLNPs from different SARS-CoV-2 variants, and then the anti-wild-type SARS-CoV-2 neutralizing antibodies in serum were detected by pseudovirus neutralization assay. NT_50_, 50% neutralization titers. N = 8 mice per group. Data are represented as the mean ± SD. Unpaired two-tailed Student’s *t* test was performed for comparison, as indicated in the figures; *P < 0.05; **P < 0.01; ***P < 0.001; ns, not significant.

To demonstrate that this CXCL13-circRNA can also help increase the breadth of antibody responses against SARS-CoV-2 variants, we tested a CXCL13-circRNA-adjuvanted SARS-CoV-2 trimeric RBD circRNA vaccine in mice (Fig. 6*A*). The results revealed that, compared to RBD-circRNA, RBD-CXCL13-circRNA promoted dLN development and GC formation (Fig. 6 *B* and *C*). We immunized mice with RBD-CXCL13-circRNA or RBD-circRNA constructed based on the Wuhan-Hu-1 sequence (Fig. 6*D*) and characterized antibody responses against the Wuhan-Hu-1 RBD, spike and S1 protein (Fig. 6*E*), and against RBD from several variants of concern (Fig. 6 *F* and *G*). We found that mice immunized with RBD-CXCL13-circRNA generated higher antibody titers and immune responses against Wuhan-Hu-1 S protein, S1 protein, RBD protein, and other mutant RBD proteins (Fig. 6 *E*–*G*). In contrast, mice immunized with RBD-circRNA generated significantly lower antibody titers against Wuhan-Hu-1 RBD protein and only partial responses (50 to 100% of immunized mice) against variants. Especially for highly contagious SARS-CoV-2 variants such as the Delta variant and Omicron variant, all mice immunized with RBD-CXCL13-circRNA induced high-titer antibodies against their RBD domains, whereas 50 to 75% of the mice immunized with RBD-circRNA generated lower antibody titers (Fig. 6*G*).

Furthermore, we adopted a pseudovirus neutralization assay to assess anti-SARS-CoV-2 neutralizing antibodies in the serum of vaccinated mice. Mice were immunized with RBD-CXCL13-circRNA or RBD-circRNA derived from various SARS-CoV-2 variants, followed by neutralization assays against wild-type pseudoviruses of the respective strains. Pseudovirus neutralization assays showed that neutralizing antibodies generated in mice immunized with RBD-CXCL13-circRNA from different viral strains were effective in neutralizing the pseudovirus (Fig. 6*H*). Regarding RBD-circRNA, apart from neutralizing antibodies produced in mice (75%) immunized with RBD-circRNA from the Wuhan-Hu-1 source, only 25 to 50% of mice generated effective neutralizing antibodies capable of neutralizing the pseudovirus for other variant-derived RBD-circRNA. In summary, these data show that the RBD-CXCL13-circRNA vaccine, constructed with CXCL13 as an immunostimulatory adjuvant, enhances both the magnitude and breadth of the antibody response against multiple SARS-CoV-2 variants.

To evaluate the immunogenic potential of the antigen-CXCL13-circRNA-tLNP vaccine system, we formulated S-mRNA-LNP vaccine similar to Moderna mRNA-1273 vaccine. The composition and ratio of lipids in the LNPs used in the S-mRNA-LNP vaccine are identical to those used in mRNA-1273, including the same ionizable lipid, SM102 (42). Both the S-CXCL13-circRNA-tLNP and S-mRNA-LNP vaccines encoded a prefusion-stabilized SARS-CoV-2 spike (S) protein, stabilized with two proline mutations (K986P and V987P) (43). The results in mice showed that both S-mRNA-LNP vaccine and S-CXCL13-circiRNA-tLNP vaccine could induce higher antibody titers after booster immunization, but IgG and NT_50_ induced by S-CXCL13-circiRNA-tLNP vaccine were significantly higher than that of S-mRNA-LNP vaccine after booster immunization (*SI Appendix*, Fig. S10). Notably, mice that received two doses of the S-CXCL13-circRNA-tLNP vaccine not only produced serum neutralizing antibodies targeting the Wuhan-Hu-1 strain but also generated neutralizing antibodies against other variants (*SI Appendix*, Fig. S10). In contrast, the S-mRNA-LNP vaccine induced relatively lower antibody titers against Wuhan-Hu-1 and showed reduced efficacy in generating antibodies against other variants.

### CXCL13 Possesses Potent Adjuvant Activity and Enhances the Efficacy of Protein Subunit Vaccines.

Protein subunit vaccines, as traditional vaccines, often exhibit relatively weak immunogenicity, requiring the addition of adjuvants to enhance the induction of immune responses (44). We have validated the successful application of CXCL13 coexpression with antigen in the circRNA vaccine platform, and now we aim to investigate whether CXCL13-circRNA exerts similar enhancing effects on protein subunit vaccines as well. Two recent studies have found that LNPs (45) and exogenous circRNA (46) can both serve as effective immunostimulatory components for mRNA vaccines, providing adjuvant effects. The adjuvant activity of tLNP-delivered CXCL13-circRNA can stem from the circRNA, from the tLNP, or from both components. To address this question, we prepared tLNPs containing either firefly luciferase (Luc)-encoding circRNA or no payload (empty tLNP, etLNP). We mixed PR8 (H1N1) influenza virus HA recombinant protein (rHA) with CXCL13-circRNA tLNPs, Luc-circRNA tLNPs, or with etLNP equivalent to the same amount of lipid, and administered them intramuscularly (i.m.) to mice (*SI Appendix*, Fig. S11*A*). The results show that compared to rHA + etLNP and rHA + Luc-circRNA, rHA + CXCL13-circRNA induced higher levels of IgG and HAI titers, broadening the antibody response against influenza virus variants (*SI Appendix*, Fig. S11 *B* and *C*). In contrast, mice immunized with rHA + etLNP and rHA + Luc-circRNA showed higher antibody responses to PR8, but only exhibited partial responses against heterologous viruses. Furthermore, the use of CXCL13-circRNA in the HA subunit vaccine exhibited a certain dose-dependent effect, with antibody responses increasing as the dose was elevated. These findings suggest that CXCL13-circRNA retains adjuvant activity in the subunit vaccine context, promoting both the magnitude and breadth of the antibody response.

To further elucidate the effectiveness of this CXCL13-circRNA formulation in inducing both the strength and breadth of antibody responses, we investigated whether CXCL13-circRNA also contributes to enhancing the breadth of the antibody response against variants of the SARS-CoV-2 RBD subunit vaccine (*SI Appendix*, Fig. S11*D*). As anticipated, CXCL13-circRNA amplified both the strength and breadth of the antibody response against variants of the SARS-CoV-2 RBD subunit vaccine (*SI Appendix*, Fig. S11 *E* and *F*). In conclusion, these data indicate that CXCL13-circRNA possesses intrinsic adjuvant properties, effectively enhancing the strength and breadth of the antibody response against viral variants, and might be a potential independent adjuvant for protein subunit vaccine platforms.

### Antibody Modification Imparts Lyophilization Stability and Targeted Stability to the LNPs.

LNP-based mRNA vaccines have been widely utilized; however, the stability of LNP-mRNA vaccines remains a significant challenge for clinical application (19, 47). To assess the thermal stability of the vaccines, the HA-CXCL13-circRNA tLNPs formulation was stored for different times (1, 7, and 14 d) at various temperatures, and changes in the basic physical properties (particle size, encapsulation efficiency, PDI) and immunogenicity of the vaccine were evaluated. The results revealed that when stored at 4 °C for 14 d, no significant changes were observed in particle size, encapsulation efficiency, PDI, or antibody titers (*SI Appendix*, Fig. S12*A*). At 25 °C (*SI Appendix*, Fig. S12*B*), the basic physical properties and antibody titers remained unaffected for at least 7 d, while storage at 37 °C (*SI Appendix*, Fig. S12*C*) resulted in a significant decrease in antibody titers by day 7. These findings indicate that the HA-CXCL13-circRNA tLNPs vaccine exhibits good short-term thermal stability after preparation.

We found that HA-CXCL13-circRNA tLNPs vaccines exhibit a certain degree of thermal stability; however, there are still significant challenges with respect to long-term storage and simplified cold chain transportation. Lyophilization is an effective method for the long-term preservation of LNP-mRNA vaccines (19). During our investigation into antibody-modified LNP targeted delivery, we found that LNPs modified with anti-DEC-205 antibody exhibit notable stability during lyophilization compared to regular LNPs. Even with the addition of sufficient sucrose (8% sucrose) (25), the physical and chemical properties of LNPs were still compromised to some extent during lyophilization, indirectly resulting in a reduction in vaccine immunogenicity (*SI Appendix*, Fig. S13*A*). In contrast, tLNPs showed no significant differences from freshly prepared vaccines in terms of their physical and chemical properties or immunogenicity (*SI Appendix*, Fig. S13*B*). Next, the postlyophilization targeting stability of tLNPs containing different concentrations of cryoprotectant (sucrose) was analyzed, and the results showed that the targeting specificity of tLNPs remained stable (*SI Appendix*, Fig. S14*A*). During this process, the sucrose content in the vaccine decreased gradually from 8% to 0%. The results reveal that when the sucrose content decreased to 2%, a slight reduction in antigen distribution of tLNPs within LNs began to occur. As the sucrose content decreased to 0%, the distribution proportion of the antigen in the LNs decreased to 23.8% of the freshly prepared formulation (*SI Appendix*, Fig. S14*B*). Further antibody data also indicate that LNPs without antibody modification exhibit poorer stability during lyophilization, which can lead to a decrease in immunogenicity (*SI Appendix*, Fig. S14*C*). In contrast, antibody-modified LNPs showed no significant difference in antibody levels compared to freshly prepared formulations when the sucrose content was 2%, only experiencing a substantial decrease when sucrose was absent (*SI Appendix*, Fig. S14*D*). Therefore, we chose the tLNPs formulation with 2% sucrose content for subsequent storage experiments.

Next, we conducted storage experiments on lyophilized samples using tLNPs containing 2% sucrose. The lyophilized samples were stored for 7 mo under conditions of either 4 °C or 25 °C (*SI Appendix*, Fig. S14*E*). The results showed that the distribution of the vaccine stored at 4 °C for 7 mo was consistent with that of the freshly prepared antigen, while the distribution ratio of LNs in the vaccine stored at 25 °C decreased significantly (*SI Appendix*, Fig. S14*F*). As expected, vaccine formulations stored at 4 °C showed a minor decrease that was not statistically significant in immunogenicity from the freshly prepared formulation after 7 mo of storage, whereas vaccine formulations stored at 25 °C exhibited a significant decline (*SI Appendix*, Fig. S14 *G* and *H*).

In summary, these experiments demonstrate that antibody-modified LNPs possess excellent lyophilization stability and targeted stability. They maintain stability for at least 7 mo in the presence of a small amount of cryoprotectant, providing a certain level of assurance for the clinical translation of the vaccine.

## Discussion

Incorporating both the antigen and adjuvant within a single vaccine formulation ensures the simultaneous delivery of these two components into the same antigen-presenting cells, demonstrating potential in the field of vaccination (10, 11). Here, we reported a general strategy to enhance the breadth and magnitude of antibody responses induced by vaccination by directly integrating both the antigen and adjuvant into the circRNA strands and coloading them within delivery carriers. Presently, we have observed that CXCL13 significantly augments both the breadth and magnitude of antibody responses against influenza and SARS-CoV-2 viruses. Furthermore, evaluations of single-dose influenza and rabies vaccines indicate that CXCL13 effectively enhances the protection of the single-dose influenza circRNA vaccine against heterologous influenza viruses and boosts the immunogenicity of the single-dose rabies circRNA vaccine, underscoring the potential of CXCL13 to facilitate the development of single-dose vaccines. HA-CXCL13-circRNA tLNPs vaccines do not necessitate supplementary exogenous materials for immune stimulation, which has potential safety advantages in clinical applications.

In recent years, an increasing amount of research has focused on using adjuvants to enhance the intensity and breadth of immune responses (48, 49). It has been reported that a polymeric Toll-like receptor 7 agonist nanoparticle (TLR7-NP) adjuvant has been shown to enhance LN targeting, resulting in persistent activation of immune cells and a broad immune response (50). Our research, along with others’, has increasingly clarified that CXCL13 not only enhances the intensity of immune responses but also has a potential association with the generation of bnAbs against influenza and HIV, among others (15–18, 51). Several groups have shown that the generation of broadly neutralizing antibodies against viruses such as HIV requires prolonged GC responses and antigen availability to modulate immune dominance and promote B cell recognition of conserved epitopes (52–55). Our results showed that GC responses were robust and exhibited a longer duration following immunization with HA-CXCL13-circRNA compared with the HA-circRNA group. In addition, the number of Tfr cells induced by HA-CXCL13-circRNA was lower. Therefore, we speculate that a low proportion of Tfr cells may be one of the reasons for the continued maintenance of GC, and robust and prolonged GC may provide the basis for B cells to recognize nondominant epitopes, increasing antibody diversity and affinity. One study indicates that IL-4 signaling controls the expansion of rare GC-B cells recognizing shared epitopes through the mTOR pathway and B cell proliferation (56). Recognition of homologous antigens presented by GC B cells by Tfh cells leads to increased expression of IL-4 and IL-21 (57). The availability of IL-21 determines the magnitude, persistence, and output of GC responses, regulating GC B cell selection and differentiation into plasma cells or memory B cells (58). IL-4 acts on B cell differentiation at multiple stages, including GC B cell and memory B cell differentiation (33). The memory response of B cells is believed to be crucial for introducing SHM into antibodies, and influenza infection appears to induce a memory response of B cells (56). Consistent with these previous findings, our results show a significant increase in the expression of IL-4 and IL-21 in mouse LN following CXCL13 immunization, accompanied by increased expansion of GC B cells and memory B cells.

Using the tLNP-circRNA system to vaccinate mice with HA-CXCL13, we found four CXCL13-mediated hallmarks: IL-21 and IL-4 production, IgG2c production, recognition of the HA stalk, and generation of cross-reactive antibodies that protect against diverse viral strains. IL-4 signaling plays a crucial role in the clonal expansion of GC-B cells, resulting in the generation of broadly protective antibodies (56). One of the reasons why CXCL13 induces broadly protective antibodies may be similar to influenza virus infection, wherein the breadth of antibody responses is expanded through IL-4 signaling in B cells (56). Studies in murine models indicate that IL-21 promotes B cell proliferation, isotype class switching, particularly to IgG (59). IgG2c is important since it proved to be the most effective isotype for influenza protection (60). In contrast to the influenza virus HA head region, the stalk region is well conserved, making it an excellent target for generating broadly cross-reactive antibodies (61). Currently, it remains unclear which factor among IL-4, IL-21, Tfh cells, and GC B cells determines the breadth of antibody response expanded by CXCL13, which may also be the result of the interaction of multiple factors.

Surface modification of LNPs provides an effective approach for targeted delivery, especially when antibodies or other molecules are conjugated to LNPs, which can enhance their targeting capability (62–64). Based on the maleimide thiol reaction, researchers prepared lung-targeted LNPs by coupling PECAM-1 antibody with LNPs, and the efficiency of LNPs in delivering mRNA in the lung was more than 200 times higher than that of their similar products (65). In this study, anti-DEC-205 antibody-modified LNPs increased antigen expression in LNs from 6.84% to 43.09%. The expression of CXCL13 in dendritic cells within the dLNs likely creates a more favorable microenvironment for the proper recruitment and activation of immune cells where they are most needed, potentially amplifying the vaccine’s effectiveness. Meanwhile, the antigen expression in the liver decreased from 16.23% to 7.81%. Targeted delivery of nanoparticles to LNs not only generates a stronger immune response but also reduces the potential liver damage caused by massive accumulation. Undesirable mRNA translation in various cells or organs resulting from off-target effects of mRNA-LNP vaccines or therapies can induce unnecessary side effects (66). Continued optimization of LNP delivery systems tailored for specific organs or cells will contribute to mitigating this concern.

Under the background of the clinical transformation of mRNA-LNP, research on improving the long-term stability of mRNA-LNP preparations has gradually attracted attention (19, 67). Some studies are evaluating the impact of lyophilization on the stability and in vivo efficacy of traditional mRNA-LNP formulations (19, 25). In this study, we found that LNPs modified by antibodies had strong stability during lyophilization, which successfully reduced the storage conditions and realized the long-term storage of the vaccine for at least 7 mo. Antibody modification retains the physicochemical properties of lyophilized LNPs and the immunogenicity of the vaccine, which is not significantly different from the freshly prepared vaccine. Currently, research in the field of lyophilization primarily focuses on exploring its impact on mRNA and LNPs formulations, with limited reports on whether lyophilization affects the surface modifications of LNPs (19, 22, 24). In this study, in addition to finding that antibody modification can improve lyophilization stability, we also found that antibody modification maintained targeted stability after lyophilization, and even in the case of containing 2% sucrose, the antigen distribution remained consistent with that of the fresh preparation. Notably, in the absence of any cryoprotectants in vaccine formulations, lyophilization has a significant disruptive effect on the surface modifications of LNPs, resulting in a pronounced decrease in targeting efficacy. This also indirectly emphasizes the critical importance of the stability of surface modifications in lyophilization for LNP vaccines that have undergone targeting modifications. We speculate that the protective effect of antibody modification on LNP lyophilization may be to adjust the freezing rate and ice crystal formation and reduce the crystal damage and liposome structure damage caused by freezing (68). Simultaneously, the antibody can be enveloped on the surface of LNPs to form a protective layer, which reduces the contact between LNPs and the surrounding environment and reduces the influence of light, oxygen, humidity, and other factors. Through these mechanisms, antibody modification can offer protection for LNP lyophilization, enhancing the stability and shelf life of LNP formulations.

Despite the success of COVID-19 vaccines, we have yet to fully exploit the potential of mRNA vaccines, and there are still some obstacles. These include inadequate targeting of organs other than the liver, insufficient breadth of antibody response, and stability issues, which are key bottlenecks in mRNA vaccine development. The intrinsic adjuvant nature of CXCL13 not only enhances the strength and breadth of the antibody response but also reduces the need for exogenous adjuvants. In addition to its potential benefits, any potential risks of side effects or adverse reactions are obstacles to the widespread use of CXCL13 as an adjuvant for circRNA vaccines. While preliminary data in mice have not indicated significant safety concerns, further research is needed to thoroughly evaluate any risks of side effects or adverse reactions. The use of antibody-targeted modifications enables the HA-CXCL13-circRNA vaccine to be delivered to the dLNs in a spatiotemporal manner to enhance immune responses while lowering the potential for adverse systemic effects. In summary, we found that the antigen-CXCL13-circRNA coexpression system based on tLNPs delivery demonstrates excellent performance in developing antibody responses against various viral variants and possesses favorable characteristics for design and long-term storage, making it highly suitable for clinical applications.

## Materials and Methods

### circRNA Synthesis.

The production of circRNAs was performed according to previous reports (27, 29). In brief, unmodified circRNA precursors were synthesized via In Vitro Transcription (IVT) from linearized circRNA plasmid templates using the T7 High Yield RNA Transcription Kit (Novoprotein, Cat. No# E131). CircRNA molecules have a covalently closed circular structure that does not require 5′-Cap protection, resulting in reduced immunogenicity and eliminating the need for nucleotide analogs. Following IVT, the RNA products were treated with DNase I (Novoprotein, Cat. No# E127) for 25 min to remove the DNA templates. Subsequently, GTP was added to the reaction at a final concentration of 2 mM, and the reactions were incubated at 55 °C for 20 min to promote circRNA cyclization. The synthesized RNA was then purified using RNA Clean Beads (Vazyme, Cat. No# N412) and heated at 65 °C for 5 min, followed by cooling on ice. Subsequently, the RNA was treated with RNase R at 37 °C for 30 min to enrich circRNAs further. RNase R (Novoprotein, Cat. No# E224) treated RNA was purified and stored at −80 °C until use. For detail, see *SI Appendix*.

### Mouse Vaccination and Serum Collection.

BALB/c or C57BL/6 mice were immunized via intramuscular (i.m.) injection with HA-circRNA, HA-CXCL13-circRNA, RBD-circRNA, or RBD-CXCL13-circRNA, and received a booster immunization with an equivalent dose 14 d after the initial immunization. Similarly, for subunit vaccine immunization, a combination of circRNA vaccines and recombinant proteins was administered, followed by a booster immunization with an equivalent dose 14 d after the primary immunization. Additionally, a separate group of mice received a single dose of each respective vaccine formulation. Serum samples were collected for antibody detection on day 14 and day 35 after prime immunization. Mice were anesthetized with isoflurane, and blood was obtained via the retro-orbital route. After a 30 min incubation at room temperature, the samples were centrifuged at 1,845×*g* for 5 min. The isolated serum samples were subsequently stored at −20 °C. All mouse sera were heat-inactivated at 56 °C for 35 min before use. For detail, see *SI Appendix*.

### Statistical Analyses.

The unpaired two-tailed Student’s *t* test, one-way ANOVA with Tukey’s post hoc test, or two-way ANOVA was performed for comparison as indicated in the figure legends. Survival statistical analysis was performed using a log-rank (Mantel–Cox) test. Statistical analyses were performed using GraphPad Prism software 9.0 (GraphPad Software, Inc., CA). The values shown in the graphs are presented as the mean ± SD. The following notations were used to indicate significant differences between groups: *P < 0.05; **P < 0.01; ***P < 0.001; ****P < 0.0001; ns, no significant difference. All *P*-values < 0.05 were considered statistically significant.

## Supplementary Material

Appendix 01 (PDF)

## Data Availability

The accession number for the raw data files for the transcriptome sequencing reported in this paper is NCBI PRJNA1118020 (69). All other data are included in the manuscript and/or *SI Appendix*.
