# Supplementary material for: CXCL13 promotes broad immune responses induced by circular RNA vaccines
Source: Proc Natl Acad Sci U S A. 2024 Oct 22;121(44):e2406434121. doi: 10.1073/pnas.2406434121 (PMC11536096; doi:10.1073/pnas.2406434121)
Supplement: Supplementary file 1 — Appendix 01 (PDF) [file pnas.2406434121.sapp.pdf]

PNAS

Supporting Information for

CXCL13 promotes broad immune responses induced by circular RNA vaccines

Jiawu Wan[1,2,3,4,†], Caiqian Wang[1,2,3,4,†], Zongmei Wang[1,2,3,4], Lingli Wang[1,2,3,4], Haoran Wang[1,2,3,4], Ming Zhou[1,2,3,4], Zhen F. Fu[1,2,3,4], Ling Zhao[1,2,3,4,*]

Correspondence to Ling Zhao

Email: zling604@outlook.com or lingzhao@mail.hzau.edu.cn

This PDF file includes:

Supporting text

Figures S1 to S14

SI References

Supporting Information Text
Supplementary Methods

Animals and ethics statement

BALB/c (6 to 8 weeks old) and C57BL/6 (5 to 7 weeks old) female mice were purchased from the Centers for Disease Control of Hubei Province, China. All mice were bred and kept under specific pathogen-free (SPF) conditions in the Laboratory Animal Center of Huazhong Agricultural University. Influenza challenge experiments were conducted within an animal biosafety level 2 (ABSL-2) facility of Huazhong Agricultural University. Any mice that experienced a reduction in their initial body weight by ≥25% were humanely euthanized and defined as dead. Other humane endpoints include signs of severe distress or suffering, such as severe lethargy, paralysis, inability to eat or drink, and severe respiratory distress. The experimental protocol was reviewed and approved by the Scientific Ethics Committee of Huazhong Agricultural University with the approval number, HZAUMO-2021-0120.

Cells and viruses

HEK-293T cells (ATCC, CRL-11268) and MDCK cells (ATCC, CCL-34) were maintained in our laboratory. The HEK293T-hACE2 cell line was obtained from Biodragon Inc. (Cat. No# BDAA0039). These cell lines were cultured in Dulbecco's modified Eagle's medium (DMEM) (Merck, Cat. No# D5546) with 10% fetal bovine serum (FBS) (Gibco, Cat. No# 16000-044), supplemented with 1% penicillin–streptomycin (Gibco, Cat. No# 15140122) in a 5% CO_2 incubator at 37 ℃. Influenza virus A/Puerto Rico/8/1934 (H1N1) was kindly donated by Dr. Hongbo Zhou (Huazhong Agricultural University, Wuhan, China) and propagated in the allantoic cavity of SPF eggs. The preparation of SARS-CoV-2 pseudovirus were performed as described previously with slight modifications (1). Briefly, the SARS-CoV-2 pseudovirions was generated through the co-transfection plasmids psPAX2, pLenti-Luc-GFP, and plasmids encoding SARS-CoV-2 S into HEK-293T cells using Lipofectamine MessengerMax (Invitrogen, Cat. No# LMRNA003). After 48 h, the supernatant containing the pseudovirus was collected, subjected to centrifugation, and filtered through a 0.45 μm sterilized membrane.

Lipid nanoparticle encapsulation of the circRNA

The LNP-circRNA vaccine encapsulated with circRNA was prepared with the LNP formula. Briefly, the circRNA was dissolved in citrate buffer (100 mM, pH 4.0). The lipid mixtures were dissolved in anhydrous ethanol at a molar ratio of 50:10:38:1.5:0.5 for ionizable lipids (SM102) (SINOPEG, Cat. No# 06040008800), 1,2-distearoyl-sn-glycero-3-phosphocholine (DSPC) (SINOPEG, Cat. No# 06030001100), cholesterol (SINOPEG, Cat. No# 06040010300), PEG-lipid (SINOPEG, Cat. No# 06020112402), and DSPE-PEG-maleimide (SINOPEG, Cat. No# 06030502202) (2). Then, the ethanol phase and the water phase were mixed in a microfluidic device at a volume ratio of 1:3. The LNP-circRNA formulations were diluted with a 35-fold volume of 1 × PBS buffer (pH 7.4), and then concentrated to an RNA concentration of 0.1 mg/ml with 30 kD Amicon Ultra Centrifugal Filters (Millipore, Cat. No# UFC903096). Finally, LNP-circRNA formulations were sterilized with a 0.22 μm filter (Millipore, Cat. No# SLGPR33RS) and stored at 4 ℃ until use.

Evaluation of encapsulation efficiency

The encapsulation efficiency of circRNA was evaluated by the Quant-iT RiboGreen RNA Assay Kit (Invitrogen, Cat. No# R11490). The standard curve of circRNA from 0 to 2000 ng/ml was prepared by continuous dilution. Triton X-100 (BioFroxx, Cat. No# 1139ML100) was diluted to 2% with 1 × TE, 100 μl was added to a 96-well black plate (Corning, Cat. No# 354717), 1 μl of prepared LNP-circRNA formulations was added, and it was treated for 5 min, then 100 μl of 200-fold diluted Quant-iT RiboGreen Reagent was added. Similarly, 100 μl of 1 × TE was added to a 96-well black plate, 1 μl of prepared LNP-circRNA formulations was added, the mixture was mixed well, and 100 μl of 2000-fold diluted Quant-iT RiboGreen Reagent was added. Measure the sample fluorescence using a fluorescence microplate reader and standard fluorescein wavelengths (excitation ~480 nm, emission ~520 nm). The encapsulation efficiency (in percentage) was calculated as (1 − (non-lysed LNPs/lysed LNPs)) × 100.

Conjugation of anti-DEC-205 antibodies to LNPs and gel filtration

The anti-DEC-205 (clone NLDC-145, RRID: AB_2281398) antibody was reduced in PBS (pH 7.4) containing 1 mM dithiothreitol (DTT) (Merck, Cat. No# 10197777001) and 5 mM ethylenediaminetetraacetic acid (EDTA) (Merck, Cat. No# E9884), with the reaction conducted at room temperature for 1 h. Then, DTT was replaced with 5 mM EDTA in PBS using 7K Zeba Spin Desalting Columns (Thermo Fisher Scientific, Cat. No# 89891). After buffer exchange, the reduced antibody was immediately added to the LNPs at a ratio of 0.53 mg antibody per ml of LNPs. The mixture was gently inverted and incubated at room temperature for 2.5 h, followed by overnight incubation at 4 ℃. After the completion of incubation, the mixture was subjected to gel filtration using Sepharose CL4B (Shanghai Yuanye Bio-Technology Co., Ltd., Cat. No# S14088) beads on a gel filtration column. The free antibody was washed away using PBS during the process. The final purified LNPs were concentrated to the desired volume using 30 kD Amicon Ultra Centrifugal Filters. By measuring the encapsulation efficiency before and after conjugation, the loss of LNPs formulations during the conjugation and gel filtration processes was calculated.

Nanoparticle characterization

Particle size (Dynamic Light Scattering, DLS) and potential measurements were conducted utilizing a Zetasizer Nano ZS apparatus (Malvern Instrument Co., Ltd.). To assess particle size and potential properties, appropriate concentrations of nanoparticles were introduced into 1 cm testing dishes and subsequently analyzed with a dynamic light scattering instrument. Transmission electron microscopy was carried out by applying 10-μl aliquots of diluted LNPs loaded with circRNA onto a glow-discharged ultrathin carbon-coated copper grid, allowing them to stand for 10 minutes (min). After removing excess liquid with filter paper, the morphology of LNPs was examined using a transmission electron microscope (HITACHI, H-7650) equipped with a field emission gun operated at 80.0 kV.

CircRNA transfection *in vitro*

HEK293T cells were seeded in 24-well plates at 2 × 10^5 per well. Subsequently, 1 μg of circRNA was transfected into HEK293T cells using Lipofectamine MessengerMax (Invitrogen, Cat. No# LMRNA003), following the manufacturer’s instructions. Six hours later, the medium was replaced with Opti-MEM™ I Reduced Serum Medium (Thermo Fisher Scientific, Cat. No# 31985070). The supernatant was collected and concentrated, and cells were lysed on ice in RIPA buffer (Thermo Fisher Scientific, Cat. No# 89900) at 48 h after transfection.

Quantification of HA expression *in vivo* or *in vitro*

HA expression was quantified using a commercial influenza A H1N1 (A/Puerto Rico/8/1934) HA detection ELISA kit (Sino Biological, Cat. No# KIT11684), following the manufacturer's guidelines. The samples were diluted or concentrated at proper ratio. This assay operates on the basis of a double-antibody sandwich principle, which allows for the detection of PR8 HA protein in samples. To briefly describe the procedure, a monoclonal antibody specific to PR8 HA protein was pre-coated onto the wells of the assay plate. Standard samples or test samples were introduced into these wells and incubated for 2 h at room temperature. Following three washes, the plates were subjected to another round of incubation with an HRP-conjugated anti-HA antibody for 1 h at room temperature. Subsequently, three additional washes were performed, and the plates were then incubated with TMB substrate (Beyotime, Cat. No# P0209). The absorbance at 450 nm was measured, and a standard curve relating absorbance at 450 nm to concentration was fitted with a linear equation to ensure accurate quantification of HA protein levels.

Biodistribution of HA protein in mice

The quadriceps muscle of the BALB/c mouse hind leg was inoculated with 15 μg of LNP-circRNA-HA or tLNP-circRNA-HA. Tissue samples from euthanized mice, heart, liver, spleen, lung, kidney, muscle, and inguinal lymph nodes, were collected at 24 h after inoculation of vaccines. The levels of HA protein in tissues were measured by an ELISA kit.

Lyophilization process

LNP-circRNA or tLNP-circRNA formulations containing different sucrose concentrations (0%, 2%, 5%, and 8% w/v) were added to penicillin bottles (West Pharma, Cat. No# 19550210) and performed lyophilization in the glass chamber of a freeze-dryer (Christ, ALPHA 2-4 LD plus). The samples were initially frozen at -45 ℃ for 2 h to solidify, followed by a primary drying cycle at -25 ℃ for 45 h and finally a secondary drying cycle at 25 ℃ for 4 h. The obtained powder was collected for storage stability study.

ELISA analysis of antibody titers

Total IgG, IgG1, and IgG2c antibodies specific for influenza HA or SARS-CoV-2 RBD were quantified using ELISA. Briefly, ELISA plates were coated with antigens (recombinant proteins) of interest (Sino Biological: H1N1 (PR8), Cat. No# 11684-V08H; H1N1 (CF09), Cat. No# 11055-V08H; H5N1 (HK03), Cat. No# 11713-V08H; H7N9 (SH13), Cat. No# 40104-V08H; H3N2 (V11), Cat. No# 40145-V08H1; H3N2 (P09), Cat. No# 40043-V08H; H9N2 (HK08), Cat. No# 40178-V08B; RBD variant (Alpha B.1.1.7), Cat. No# 40592-V02H1); RBD variant (Beta B.1.351), Cat. No# 40592-V08H59); RBD variant (Gamma P.1), Cat. No# 40592-V02H5); RBD variant (Lambda C.37), Cat. No# 40592-V08H113); RBD variant (Delta B.1.617.2), Cat. No# 40592-V08H90); RBD variant (Omicron B.1.1.529), Cat. No# 40592-V05H3); RBD variant (Omicron XBB.1.5), Cat. No# 40592-V08H146); RBD variant (Omicron EG.5.1), Cat. No# 40592-V08H151). Vazyme: Wuhan-Hu-1 strain S, Cat. No# CG202); Wuhan-Hu-1 strain S1, Cat. No# CG219); Wuhan-Hu-1 strain RBD, Cat. No# CG201) (3 μg/ml, 50 μl) diluted coating buffer, which was coated overnight at 4 ℃. The plates were subjected to a 1 h blocking step using blocking buffer, followed by washing and incubation with diluted mouse sera for 2 h at 37 ℃. Plates were then washed and incubated for 1 h at 37 ℃ with horseradish peroxidase (HRP)-conjugated anti-mouse IgG (RRID: AB_2904507), IgG1 (RRID: AB_2769852), and IgG2c

(RRID: AB_2794462) antibodies. Following incubation, plates were washed again and developed with tetramethylbenzidine (TMB) substrates (Solarbio, Cat. No# PR1200) (100 μl/well) for 15 min. The reaction was then stopped by adding 50 μl of 2 M H_2SO_4. Optical densities (OD) were measured at 450 nm using a SpectraMax 190 spectrophotometer (Molecular Devices, CA, USA). The IgG, IgG1 and IgG2c endpoint titer was defined as the dilution fold.

Hemagglutination Inhibition (HAI) Assay

The HAI assay was used to determine the titers of HA specific functional antibodies in serum samples. Initially, these serum samples were diluted 1:20 in PBS, followed by serial 1:2 dilutions in a 96-well V-bottom plate (Thermo Fisher Scientific, Cat. No# 2605). At room temperature, serial 2-fold dilutions of heat-inactivated serum samples were incubated with an equal volume of four agglutinating doses of influenza virus A/Puerto Rico/8/1934 (H1N1). Subsequently, 1% (v/v) chicken erythrocytes (Sbjbio, Cat. No# SBJ-RBC-C001) were added, and the mixture was gently combined with the serum-virus solution. The samples were then incubated for 40 min at room temperature. After incubation, the plates were gently tilted for one minute, and the formation of a "tear-drop" shape was observed. HAI titers were defined as the highest dilution of the sample that completely inhibited red blood cell hemagglutination.

Pseudovirus neutralization assay

SARS-CoV-2 neutralizing antibody titers were tested as described previously with slight modifications (3). Briefly, to determine the 50% neutralization titer (NT_{50}) of immunized mouse serum, HEK293T-hACE2 cells were seeded in 96-well plates at a density of 40,000 cells per well and incubated for approximately 24 h until they reached over 90% confluence in preparation for SARS-CoV-2 pseudovirus infection. Subsequently, serially diluted serum samples were incubated with SARS-CoV-2 pseudovirus for 1 h at 37 ℃ and then added to the 96-well plates. The cells were further incubated for 21-24 h in a 5% CO_2 environment at 37 ℃. A negative control using DMEM was included for comparison. Following the incubation period, the supernatant was removed, and a luciferase substrate was added to each well. The mixture was then incubated for 2 min in the dark at room temperature. Luciferase activity was measured using a GloMax® 96 Microplate Luminometer (Promega). Alternatively, after incubation period, GFP expression levels were observed using Olympus IX51 fluorescence microscopy (Olympus, Tokyo, Japan) to assess effective neutralization of the pseudovirus. The NT_{50} was defined as the fold dilution that achieved more than 50% inhibition of pseudovirus infection compared to the control group.

Flow cytometry (FCM)

To identify Tfh cells, GC B cells, and MBCs, inguinal lymph nodes were harvested and homogenized into single-cell suspensions using a syringe plunger and passed through a 40 μm nylon filter (SPL Life Sciences Co., Ltd). After washing two times, single-cell suspensions containing 1×10^6 cells were blocked in PBS buffer with 0.2% bovine serum albumin (BSA). Next, the cells were stained separately with cocktails of the following fluorescently labeled antibodies: CD3 (clone 145-2C11, eBioscience), CD4 (clone GK1.5, eBioscience), CD8 (clone 53-6.7, Biolegend), B220 (clone RA3-6B2, eBioscience), CD279 (PD-1) (clone RMP1-30, Biolegend), CD185 (CXCR5) (clone L138D7, Biolegend), GL7 (clone GL7, Biolegend), CD95 (Fas) (clone SA367H8, Biolegend), CD38 (clone 90, Biolegend), and IgD (clone 11-26c.2a,

Biolegend). For HA-specific GC B cells and MBCs quantitation, recombinant His-HA protein was biotinylated by using a Biotinylation Kit (Genemore, Cat. No# G-MM-IGT). Single-cell suspensions were incubated with 5 μg/mL His-HA-Biotin for 30 min and washed. The cells were then incubated with PE Streptavidin (Biolegend, Cat. No# 405203) for 30 min prior to FCM analysis. Live single cells were identified by 7-AAD Viability Staining Solution (Biolegend, Cat. No# 420404).

To identify Tfr cells, single-cell suspensions were washed two times with PBS and stained with Zombie Aqua™ Fixable Viability Kit (Biolegend, Cat. No# 423101). Then, cells were washed, blocked, and stained with surface markers including CD3 (clone 145-2C11, eBioscience), CD4 (clone GK1.5, eBioscience), CD8 (clone 53-6.7, Biolegend) B220 (clone RA3-6B2, eBioscience), CD279 (PD-1) (clone RMP1-30, Biolegend). After staining, cells were washed and fixed using True-Nuclear™ Transcription Factor Buffer Set (Biolegend, Cat. No# 424401) according to the manufacturer's instructions. Cells were then washed, permeabilized, and stained for intracellular markers, including BCL6 (clone 7D1, Biolegend), FOXP3 (clone MF-14, Biolegend). Cells were collected using Cytek Aurora/NL and data were analyzed by FlowJo software V_10.

Intracellular cytokine staining (ICCS)

To identify antigen-specific T cells, freshly isolated splenocyte suspensions were stimulated with 10% RPMI (negative control, Gibco) or 10–30 μg/ml homologous HA protein for 18 h (37 ℃, 5% CO_2). Golgi Stop and Golgi Plug (BD Biosciences) were added 6 h before the end of the stimulation according to the manufacturer's instructions. Cell suspensions were washed two times with PBS and stained with Zombie Aqua™ Fixable Viability Kit (Biolegend, Cat. No# 423101). Then, cells were washed, blocked, and stained with surface markers including CD3 (clone 145-2C11, eBioscience), CD4 (clone GK1.5, eBioscience), CD8 (clone 53-6.7, Biolegend), B220 (clone RA3-6B2, eBioscience), CD44 (clone IM7, Biolegend), CD62L (clone MEL-14, Biolegend). After washing two times with PBS, the cells were performed with a FluoroFix™ Buffer (Biolegend, Cat. No# 422101) for 20 min at RT in the dark and permeabilizated with intracellular staining perm wash buffer (Biolegend, Cat. No# 423101). Subsequently, the cells were incubated for intracellular staining with the following antibodies (30 min, 4 ℃): IL-2 (clone JES6-5H4, Biolegend), TNF-α (clone MP6-XT22, Biolegend), IFN-γ (clone XMG1.2, Biolegend). Cell sorting was performed with Cytek Aurora/NL and data were analyzed by FlowJo software V_10.

Influenza viral challenge

The influenza virus A/Puerto Rico/8/1934 (H1N1) challenge model has been characterized in detail with slight modifications (4). After 49 days of primary vaccination, BALB/c mice were anesthetized using isoflurane and then intranasally administered 50 μl of influenza virus A/Puerto Rico/8/1934 (H1N1) containing 4×10^5 plaque-forming units (PFU). The mouse body weight and survival were monitored within 12 days post-infection. Mice that lost ≥25% of their initial body weight were euthanized and defined as dead. Another group of mice was euthanized on the fifth day after infection for viral detection and histopathological analysis.

Influenza viral plaque assay

Following infection, lungs were harvested from mice and individually homogenized in 1 ml DMEM, then stored at -80 °C until virus quantification was performed. Briefly, one day before

infection, MDCK cells were seeded in 12-well plates at a density of 1.5×10^5 cells per well. The next day, remove the culture medium and wash the cells with PBS. Subsequently, 1 ml of the sample dilution was added to each well. The plates were then incubated at 37 ℃ with 5% CO_2 for 1 h, with gentle shaking every 15 min. After incubation, the virus inoculum was removed, and the cells overlaid with DMEM containing 1.6% agarose solution and 2 μg/ml L-(tosylamido-2-phenyl) ethyl chloromethyl ketone (TPCK)-treated trypsin (Sigma). The plates were then further incubated at 37 ℃ with 5% CO_2 for 48 h. Following incubation, the cells were fixed with 4% (v/v) paraformaldehyde solution and stained with 0.25% w/v crystal violet. Plaque counting was performed, and the titer was calculated.

Histopathology assay

For histopathology, lung tissue samples were obtained from euthanized mice. These lung tissues were fixed in a 4% (v/v) paraformaldehyde solution for duration of 48 h to ensure proper fixation. Subsequently, thin paraffin slides, approximately 3~4 μm in thickness, were prepared from the samples. Hematoxylin and eosin (H&E) staining was applied to these slides to facilitate the identification of histopathological changes in the tissues. The assessment of lung tissue lesions was based on the degree of alveolar septal thickening, alveolar congestion/edema, inflammatory cell infiltration, bronchial/bronchiolar inflammation, type II pneumocyte hyperplasia, endothelial cell vacuolation, and other indicators. Histopathological were graded on a six-point scale according to severity: normal (0), minimal (1-2), mild (3-4), moderate (5-6), marked (7-8), and severe (9-10).

Immunofluorescence assay

Lung tissue paraffin sections were initially deparaffinized in xylene, rehydrated through a series of ethanol/water solutions, and then treated with 3% H_2O_2 at room temperature. Subsequently, the sections underwent antigen retrieval at 95 ℃ for 1 h in 10 mM sodium citrate buffer (pH = 6). A 30-minute blocking step with saturated BSA followed. The primary antibody (Sino Biological, Cat. No# 11684-MM03) against influenza virus A/Puerto Rico/8/1934 (H1N1) HA protein was then incubated for 2.5 h at 37 ℃ within a humidified chamber, and detection was carried out using a secondary antibody conjugated with FITC. For imaging the distribution of HA and CXCL13, inguinal lymph nodes were collected for cryosectioning at 11h, 20h, 24h, and 30h after intramuscular injection of HA-CXCL13-circRNA tLNP. Sections were blocked in PBS supplemented with 10% concentrated goat serum (Boster) for 2 h at room temperature and stained with anti-His Tag (clone J095G46, Biolegend), anti-mouse DYKDDDDK Tag (clone FG4R, eBioscience), anti-mouse CD11c (clone N418, eBioscience), and 4,6-diamidino-2-phenylindole (DAPI). For germinal center staining, sections of dLN were blocked in PBS supplemented with 10% concentrated goat serum for 2 h at room temperature and stained with anti-mouse/human CD45R/B220 (clone RA3-6B2, Biolegend) and anti-mouse GL7 (clone GL7, Biolegend). Images were captured using a BX63 fluorescence microscope (Olympus, Tokyo, Japan) and processed using Image-Pro Plus 6.0 software.

Transcriptome sequencing

Total RNA was isolated from inguinal lymph nodes using TRIzol reagent (Invitrogen) according to the manufacturer’s instructions. Bioanalyzer 2100 System (Agilent Technologies) was used to evaluate the integrity and amounts of RNA samples. First, mRNA is enriched from the total RNA using magnetic beads with Oligo (dT). Fragmentation buffer is then added to break the

mRNA into short fragments. Using mRNA as a template, the first-strand cDNA is synthesized with random hexamers. Buffer, dNTPs, RNase H, and DNA polymerase I are added to synthesize the second-strand cDNA. The double-stranded cDNA undergoes end repair and addition of an A-tail at the 3' end. Sequencing adapters are ligated, and the products are purified and size-selected using Hieff NGS® DNA Selection Beads. The adapter-ligated products are then PCR amplified for enrichment. The double-stranded target region library is denatured, circularized, and digested to obtain single-stranded circular DNA. The single-stranded circular DNA is amplified through rolling circle amplification, producing DNA Nano Balls (DNBs). After library construction, the quality of the library is assessed. The prepared DNBs are loaded onto a Patterned Array for sequencing, where sequencing primers and fluorescent probes are anchored to the DNBs. High-resolution imaging captures, reads, and identifies the light signals to obtain single-base sequence information, which, after multiple cycles, yields the raw sequencing data. Sequencing service was provided by Bioyi Biotechnology Co., Ltd. Wuhan, China.

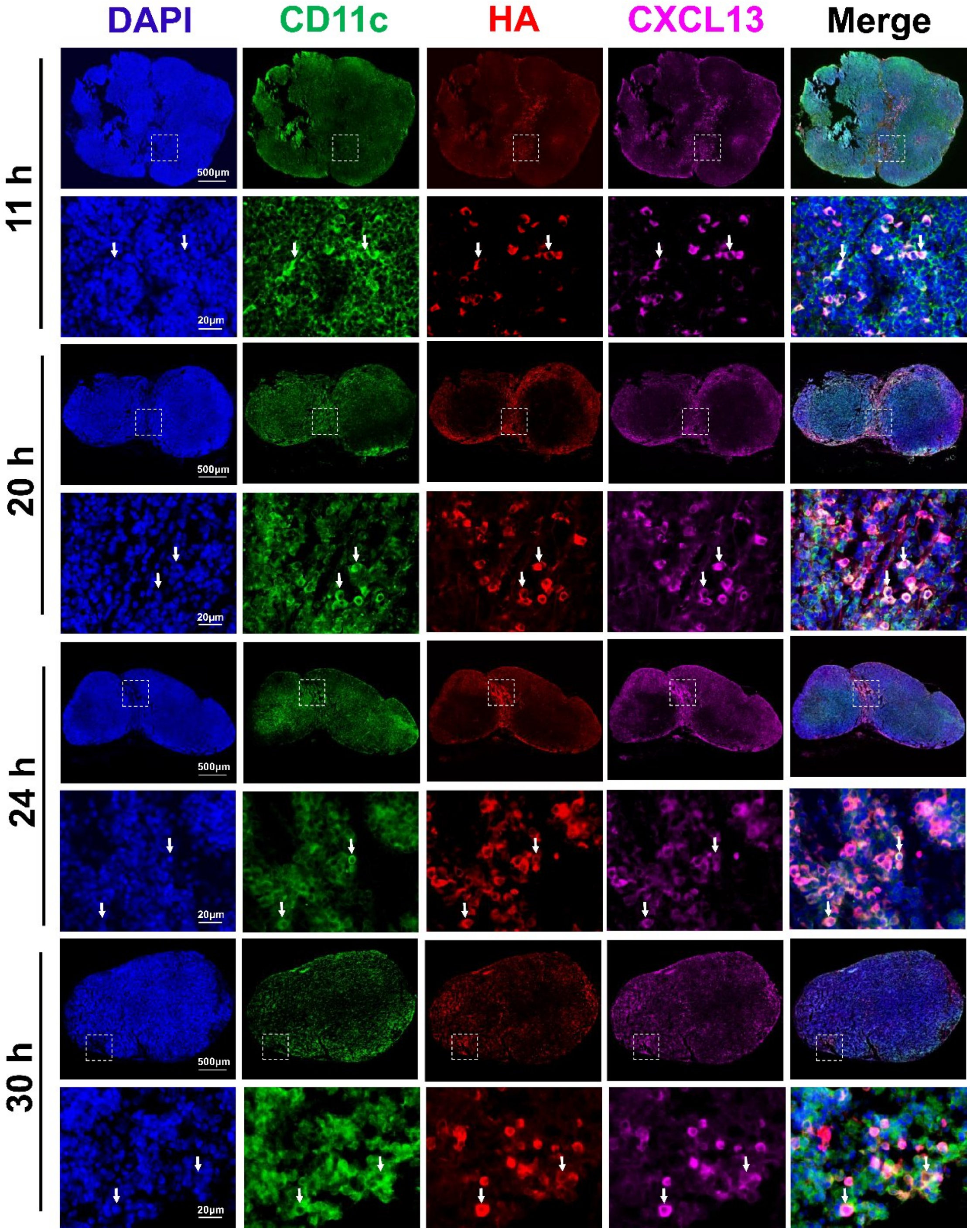

Fig. S1. Multiplex immunostaining analysis for expression of tLNP-delivered HA-CXCL13-circRNA in mouse draining lymph nodes. C57BL/6 mice (n = 3) were immunized with 5 μg of HA-CXCL13-circRNA tLNPs. Inguinal lymph nodes were collected at 11, 20, 24, and 30 hours post-injection and subjected to multi-color immunofluorescence staining for HA (red), CXCL13 (magenta), and CD11c (green). The magnified images below show the boxed areas in white. Arrows indicate positive-stained cells.

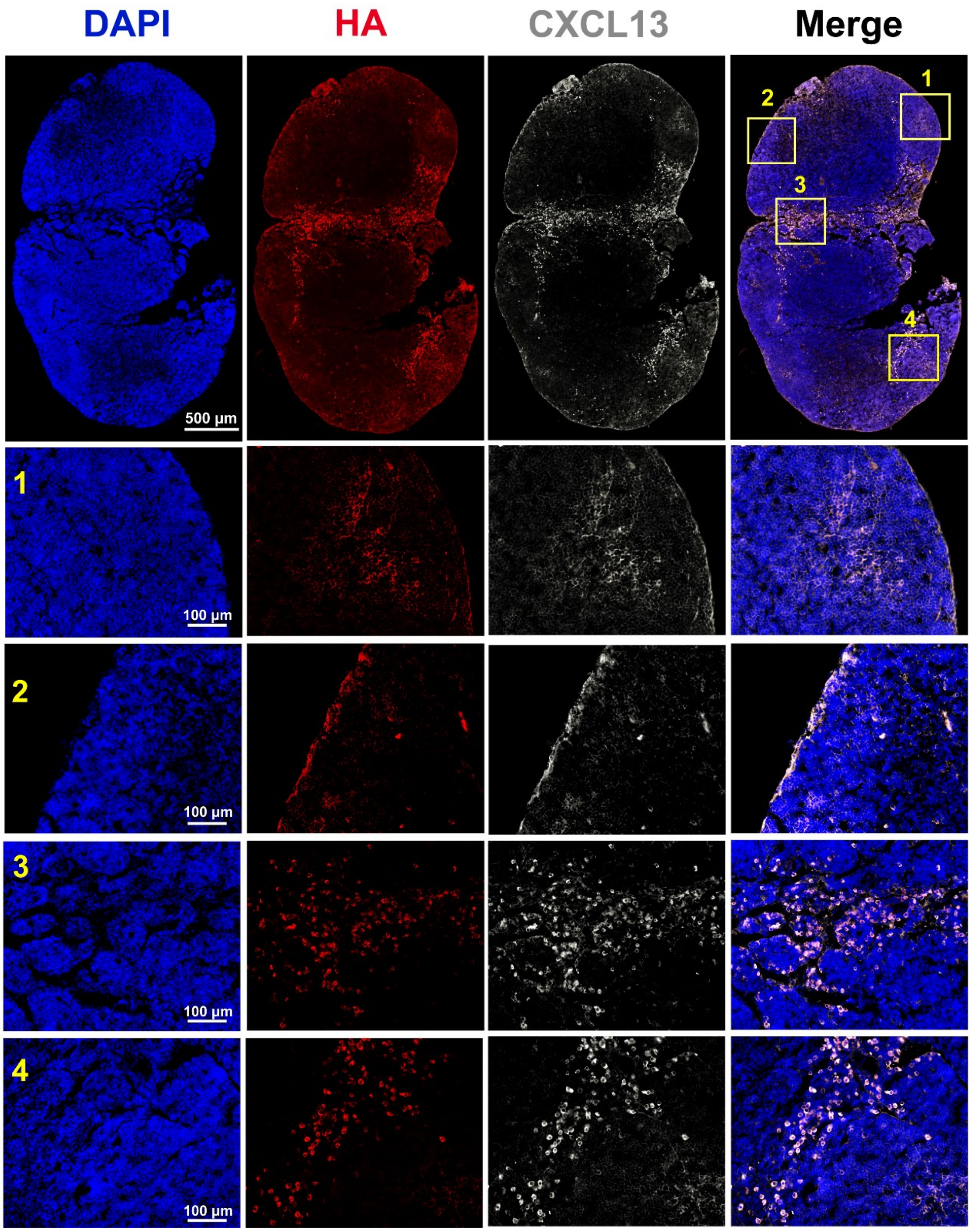

Fig. S2. Distribution of HA antigen and CXCL13 in inguinal lymph nodes. C57BL/6 mice were intramuscular injection with 5 µg of HA-CXCL13-circRNA tLNPs. Inguinal lymph nodes were collected at 11 hours post-injection and subjected to multi-color immunofluorescence staining for HA (red), CXCL13 (gray), and DAPI (blue). Four representative areas of interfollicular region, subcapsular sinus, and medulla are shown in magnification.

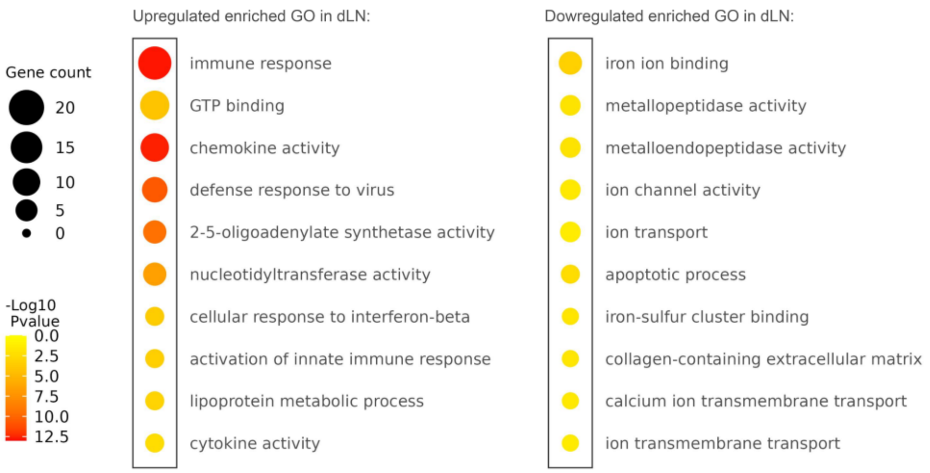

Fig. S3. Transcription profiling of mouse LNs inoculated with CXCL13. GO enrichment analysis of differentially expressed genes. The data are represented as circles, where the size indicates the gene count for that particular process, and the color represents the $-\text{Log}_{10}$ *p*-value calculated with one-sided Fisher's Exact test with Benjamini–Hochberg correction. GO: Gene Ontology.

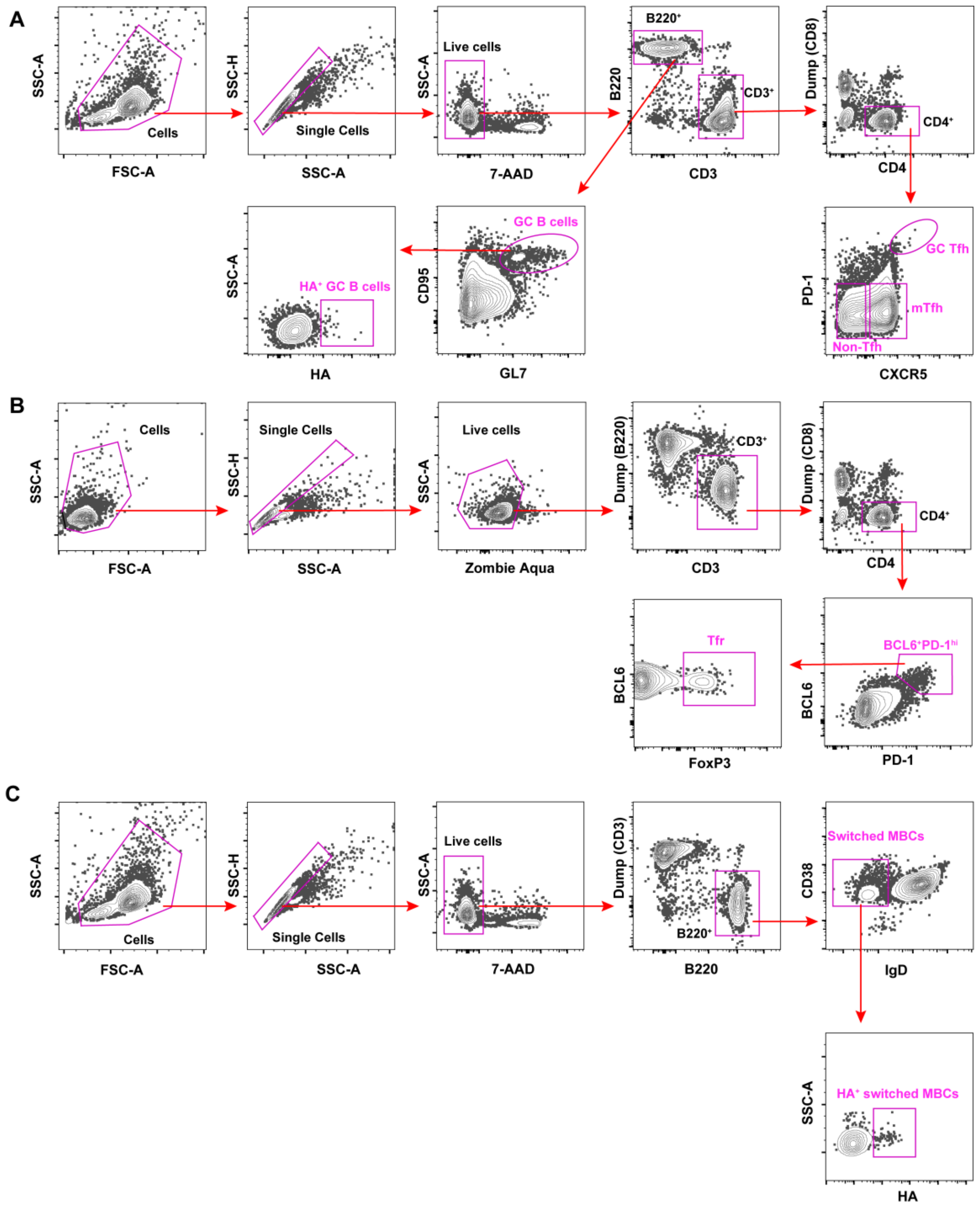

Fig. S4. Flow cytometry gating strategy for GC B cells, Tfh cells, Tfr cells and memory B cells. Cells were first gated to exclude debris and dead cells. (A) GC B cells were gated on $CD3^-B220^+CD95^+GL7^+$ cells. HA-specific GC B cells were gated on $CD3^-B220^+CD95^+GL7^+HA^+$ cells. GC Tfh cells were gated on $B220^-CD3^+CD8^-CD4^+CXCR5^{hi}PD\text{-}1^{hi}$ cells, mTfh cells were gated on $B220^-CD3^+CD8^-CD4^+CXCR5^{int}PD\text{-}1^{int}$, and non-Tfh cells were gated on $B220^-CD3^+CD8^-CD4^+CXCR5^-$. (B) Tfr cells gated on $B220^-CD3^+CD8^-CD4^+PD\text{-}1^+BCL6^{hi}FoxP3^+$. (C) HA-specific switched MBCs were gated on $CD3^-B220^+IgD^-CD38^+$ HA^+ cells.

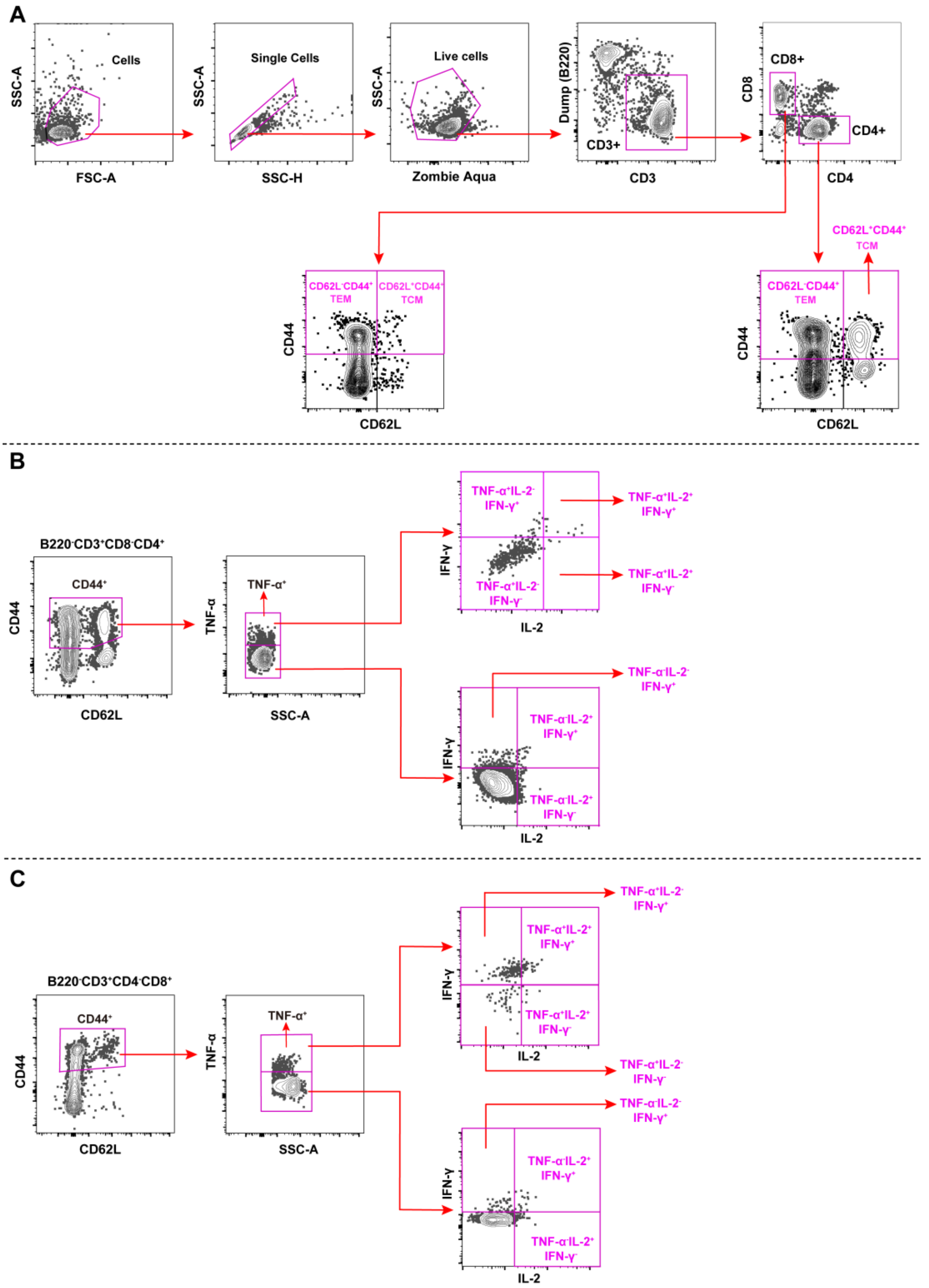

Fig. S5. Flow cytometry gating strategy to identify TEMs, TCMs and antigen-specific CD4$^+$ T cells and CD8$^+$ T cells. Cells were first gated to exclude debris and dead cells. (A) Gating strategy to identify CD4$^+$ TEMs (gated on B220$^-$CD3$^+$CD8$^-$CD4$^+$CD44$^+$CD62L$^-$) and TCMs (gated on B220$^-$CD3$^+$CD8$^-$CD4$^+$CD44$^+$CD62L$^+$). CD8$^+$ TEMs were gated on B220$^-$CD3$^+$CD4$^-$CD8$^+$CD44$^+$CD62L$^-$ and TCMs were gated on B220$^-$CD3$^+$CD4$^-$CD8$^+$CD44$^+$CD62L$^+$. (B-C) Antigen experienced CD4$^+$ or CD8$^+$ T cells were distinguished based on the expression of CD44. HA-specific multifunctional CD4$^+$ (B) and CD8$^+$ (C) T cells were further identified based on the expression of TNF-α, IL-2 and/or IFN-γ.

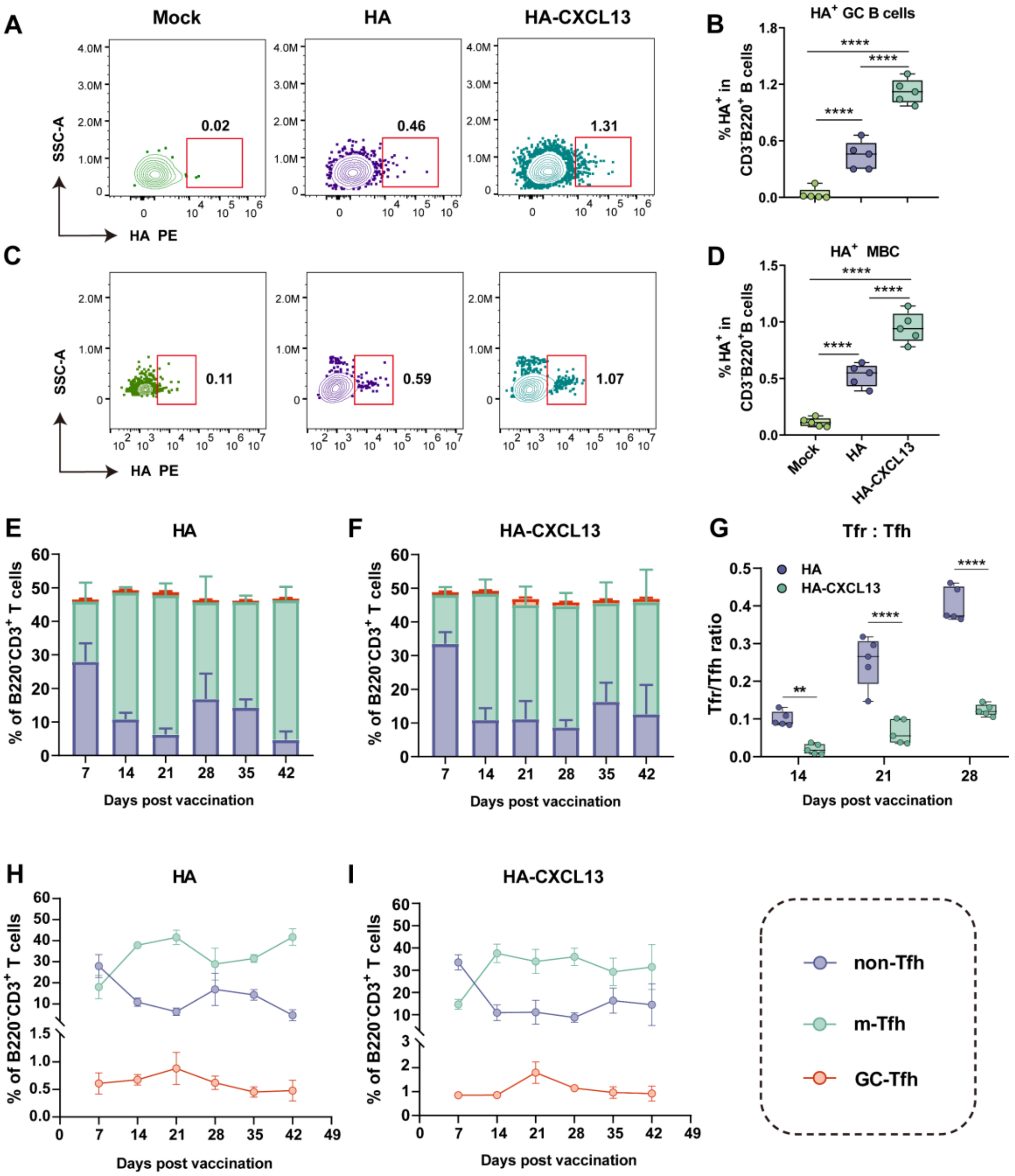

Fig. S6. HA-specific B cell response after HA-CXCL13-circRNA immunization. (A) Representative flow cytometric plots show the frequencies of HA-specific GC B cells (gated on $CD3^-B220^+CD95^+GL7^+HA^+$ cells). (B) Statistical results of HA-specific GC B cell (n = 5 mice per group). (C) Representative flow cytometric plots of HA-specific switched MBCs (gated on $CD3^-B220^+IgD^-CD38^+HA^+$ cells). (D) Statistical results of HA-specific switched MBCs (n = 5 mice per group). (E-F) Constitution of the $CD4^+$ T cell populations (GC Tfh, mTfh, and non-Tfh) of inguinal lymph nodes in HA-circRNA group (E) and HA-CXCL13-circRNA group (F). (G) The ratio of Tfh and Tfr cell numbers (n = 5 mice per group). (H-I) The frequencies of GC Tfh, mTfh, and non-Tfh cells in HA-circRNA group (H) and HA-CXCL13-circRNA group (I) at the indicated time points (n = 5 mice per group). Data are represented as the mean ± S.D. Statistical significance was determined by one way or two-way ANOVA with Tukey's multiple comparisons (**$p < 0.01$, ****$p < 0.0001$).

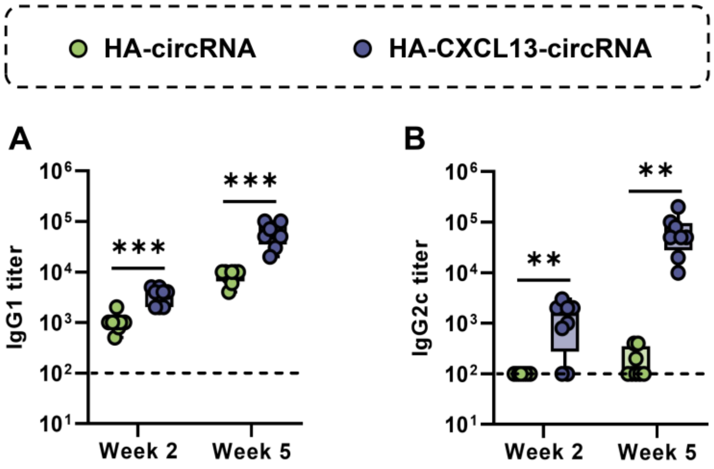

Fig. S7. Analysis of IgG subtypes specific to HA. C57BL/6 mice were injected with HA-circRNA tLNPs and HA-CXCL13-circRNA tLNPs vaccines (n = 8). Perform serological analysis of specified PR8 (H1N1) HA IgG subtypes titers by ELISA. Data are represented as the mean ± S.D. Unpaired two-tailed Student's *t* test was performed for comparison, as indicated in the figures; $^{**}p < 0.01$; $^{***}p < 0.001$.

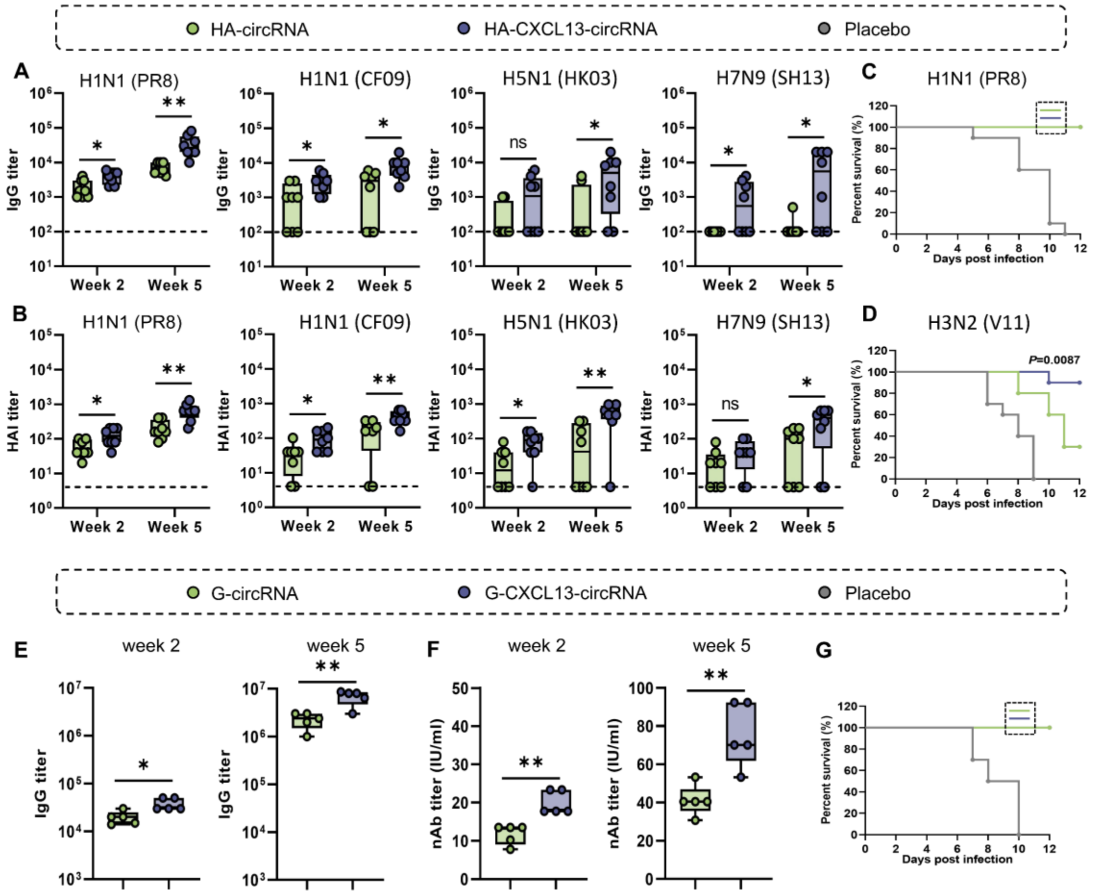

Fig. S8. The protective efficacy of a single dose of HA-CXCL13-circRNA or G-CXCL13-circRNA against influenza virus or rabies virus infection in mice, respectively. (A) BALB/c mice (n = 8) were immunized with a single intramuscular injection of 10 μg HA-circRNA tLNPs or HA-CXCL13-circRNA tLNPs, with the vaccine based on the HA sequence from the PR8 (H1N1). Serum samples were collected at weeks 2 and 5 post-immunization to measure IgG responses against the indicated HA proteins (PR8, CF09, HK03 and SH13). (B) For HAI assays, vaccines were constructed using HA sequences from PR8, CF09, HK03, and SH13 strains. Serum was collected at weeks 2 and 5 post-immunization to detect HAI titers against PR8 (H1N1) virus. (C) Mice (n = 10) were immunized with a single intramuscular injection of 10 μg HA-circRNA tLNPs or HA-CXCL13-circRNA tLNPs vaccine, with the vaccine based on the HA sequence from the PR8 (H1N1). On day 35 post-immunization, the mice were intranasally infected with PR8 (H1N1) virus, and survival was recorded for 12 days. (D) Mice (n = 10) were immunized with a single intramuscular injection of 10 μg HA-circRNA tLNPs or HA-CXCL13-circRNA tLNPs vaccine, with the vaccine based on the HA sequence from the H3N2 (V11). On day 35 post-immunization, the mice were intranasally infected with PR8 (H1N1) virus, and survival was recorded for 12 days. (E-G) BALB/c mice (n = 5) were vaccinated with a single intramuscular injection of 2 μg G-circRNA tLNPs or G-CXCL13-circRNA tLNPs vaccine. Sera were collected at weeks 2 and 5 post-immunization for IgG and neutralizing antibody (nAb) titers detection. Another group of mice (n = 10) was infected intracranially with rabies virus (50 LD_{50} of CVS-24) on day 21 post-immunization, and survival was recorded for 12 days. Data are represented as the mean ± S.D. Unpaired two-tailed Student's *t* test was performed for comparison, as indicated in the figures; *$p < 0.05$; **$p < 0.01$; ns, not significant.

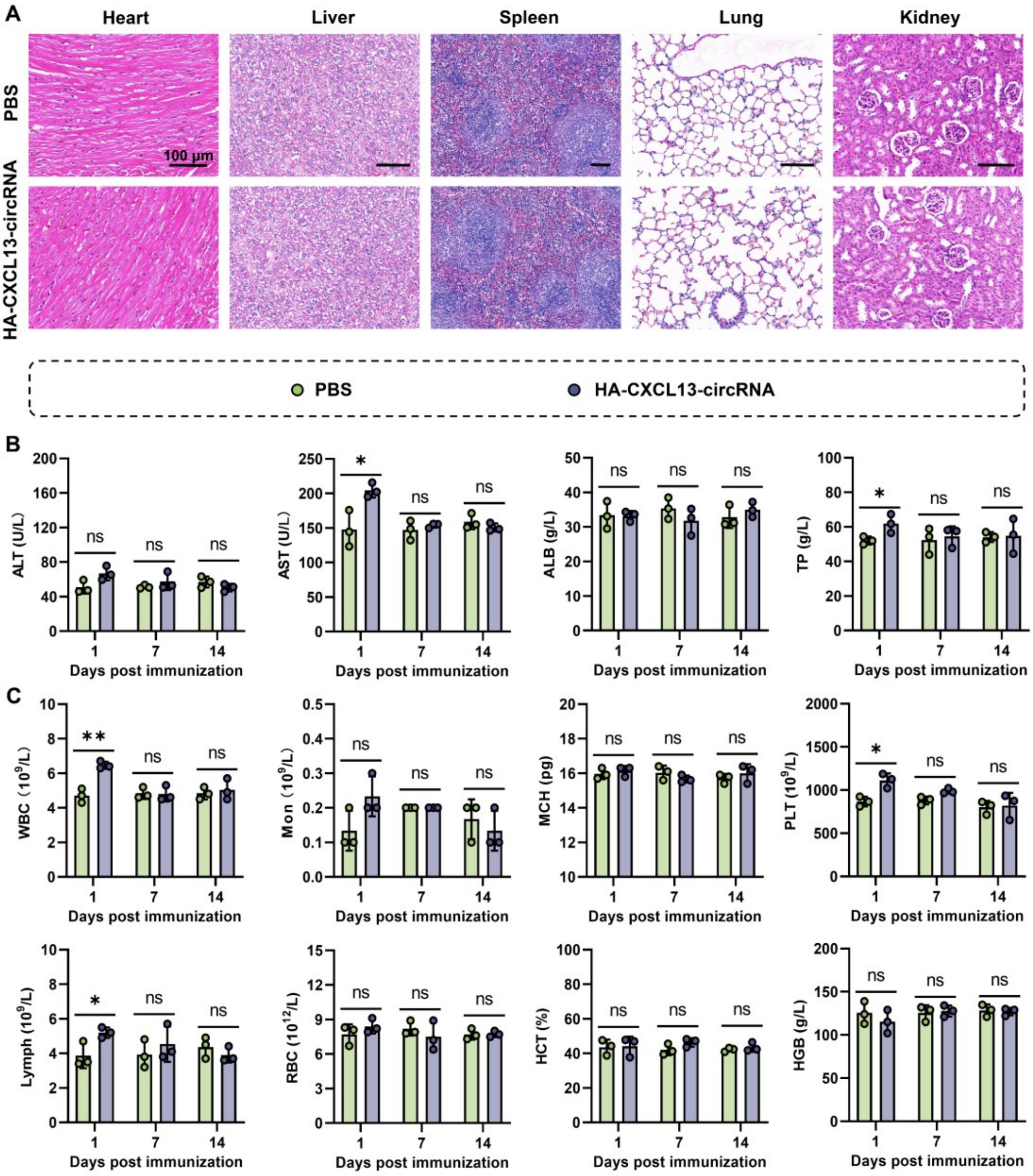

Fig. S9. Safety evaluation of HA-CXCL13-circRNA vaccines. BALB/c mice (n = 3) were intramuscular injected with 100 μl of PBS or 100 μg of HA-CXCL13-circRNA tLNPs vaccines. Blood was collected for analysis on the 1st, 7th and 14th day, and major organs were collected for H&E analysis on the 14th day. (A) Representative H&E staining images of major organs (heart, liver, spleen, lung, and kidney) of PBS- or HA-CXCL13-circRNA vaccines-injected mice. Scale bar = 100 μm. (B) Hepatic and renal functional biomarkers in the blood samples collected from the mice on day 7, 14 and 28 after subcutaneous administration of PBS and HA-CXCL13-circRNA vaccines. (C) Blood was collected at days 1, 7 and 14 for hematological analysis. Data are represented as the mean ± S.D. Unpaired two-tailed Student's *t* test was performed for comparison, as indicated in the figures; *$p < 0.05$; **$p < 0.01$; ns, not significant.

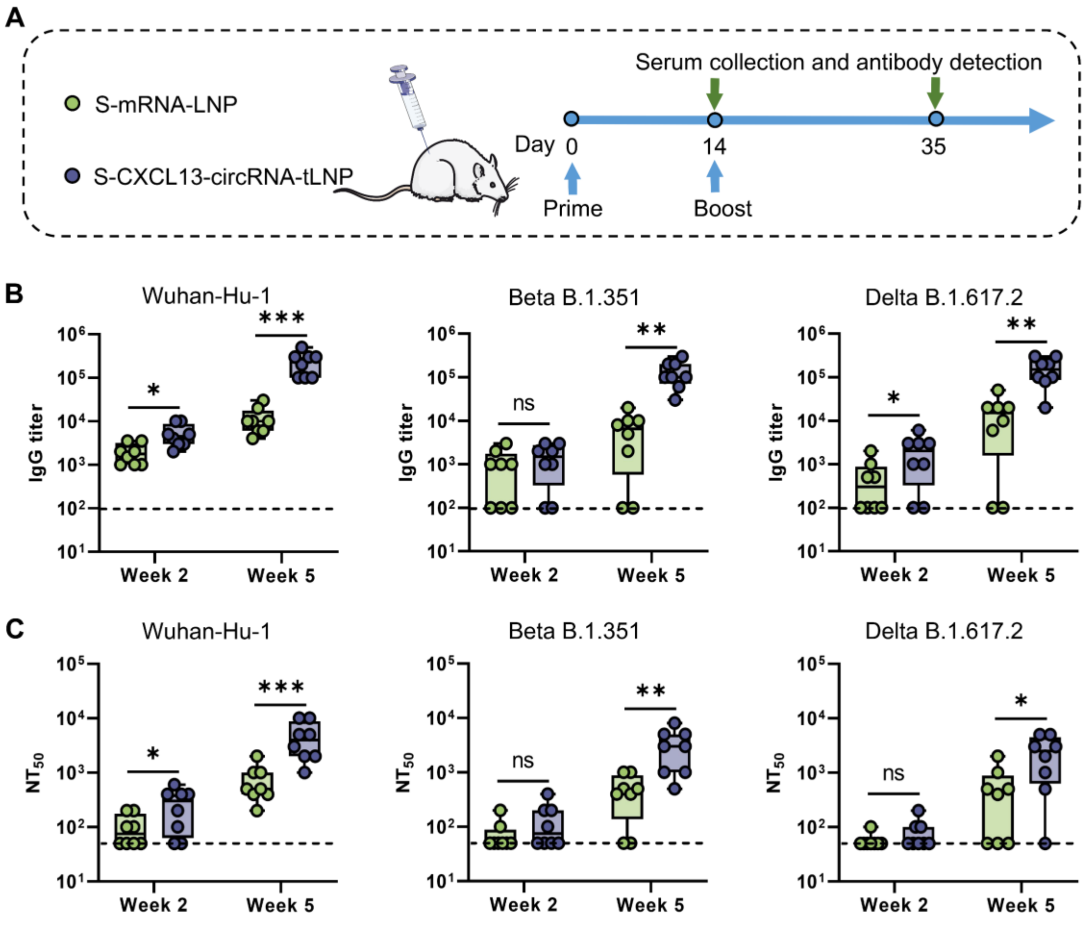

Fig. S10. S-CXCL13-circRNA tLNP immunization elicits a humoral immune response in mice. BALB/c mice (n = 8) were immunized i.m. with 10 µg of S-CXCL13-circRNA tLNP or S-mRNA LNP and boosted with an equivalent dose 14 days later. Serum was collected 14 and 35 days after initial vaccination. (A) Schematic diagram of immunization and sample collection. (B) The SARS-CoV-2-specific IgG antibody titer was determined by ELISA. (C) NT_{50} were determined using SARS-CoV-2 pseudovirus. NT_{50}, 50% neutralization titers. Data are represented as the mean ± S.D. Unpaired two-tailed Student's *t* test was performed for comparison, as indicated in the figures; *$p < 0.05$; **$p < 0.01$; ***$p < 0.001$; ns, not significant.

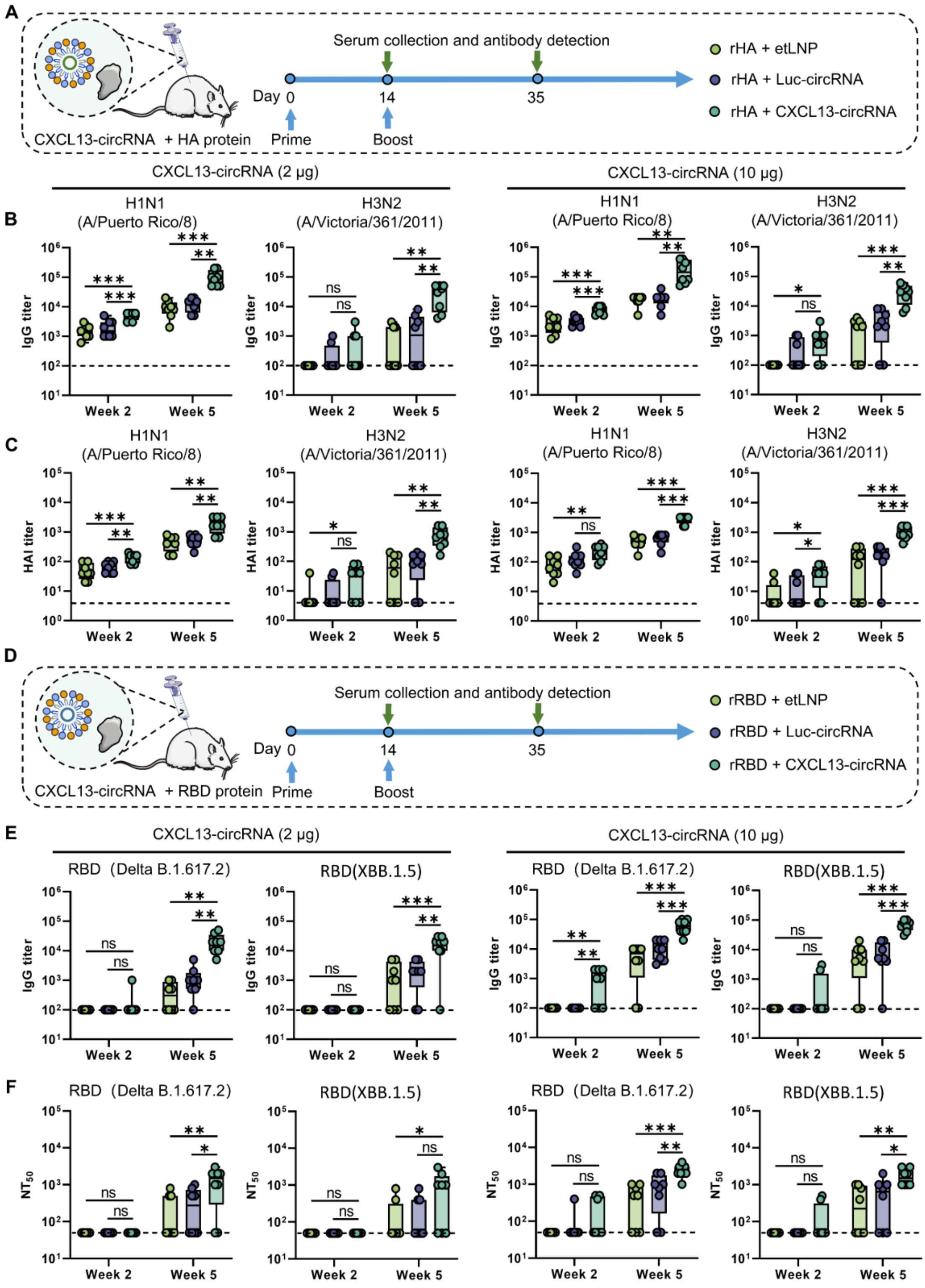

Fig. S11. Adjuvant effect of CXCL13-circRNA on protein subunit vaccines. (A) Schematic diagram of the rHA/CXCL13-circRNA formulation as a combination of rHA protein and CXCL13-circRNA tLNPs. BALB/c mice received two intramuscular immunizations with 10 μg of rHA mixed with etLNP (equal amount of tLNP as in 2 or 10 μg of CXCL13-circRNA tLNPs), Luc-circRNA tLNPs (2 or 10 μg of Luc-circRNA tLNPs) or CXCL13-circRNA tLNPs (2 or 10 μg of CXCL13-circRNA tLNPs). Serum samples were collected at 14 and 35 days after the prime

immunization for IgG (B) or HAI (C) detection. (D) Schematic diagram of the rRBD/CXCL13-circRNA formulation as a combination of rRBD protein and CXCL13-circRNA tLNPs. BALB/c mice received two intramuscular immunizations with 10 μg of rRBD mixed with etLNP (equal amount of tLNP as in 2 or 10 μg of CXCL13-circRNA tLNPs), Luc-circRNA tLNPs (2 or 10 μg of Luc-circRNA tLNPs) or CXCL13-circRNA tLNPs (2 or 10 μg of CXCL13-circRNA tLNPs). Serum samples were collected at 14 and 35 days after the prime immunization for IgG (E) or NT_{50} (F) detection. N = 8 mice per group. Data are represented as the mean ± S.D. Unpaired two-tailed Student's *t* test was performed for comparison, as indicated in the figures; $*p < 0.05$; $**p < 0.01$; $***p < 0.001$; ns, not significant.

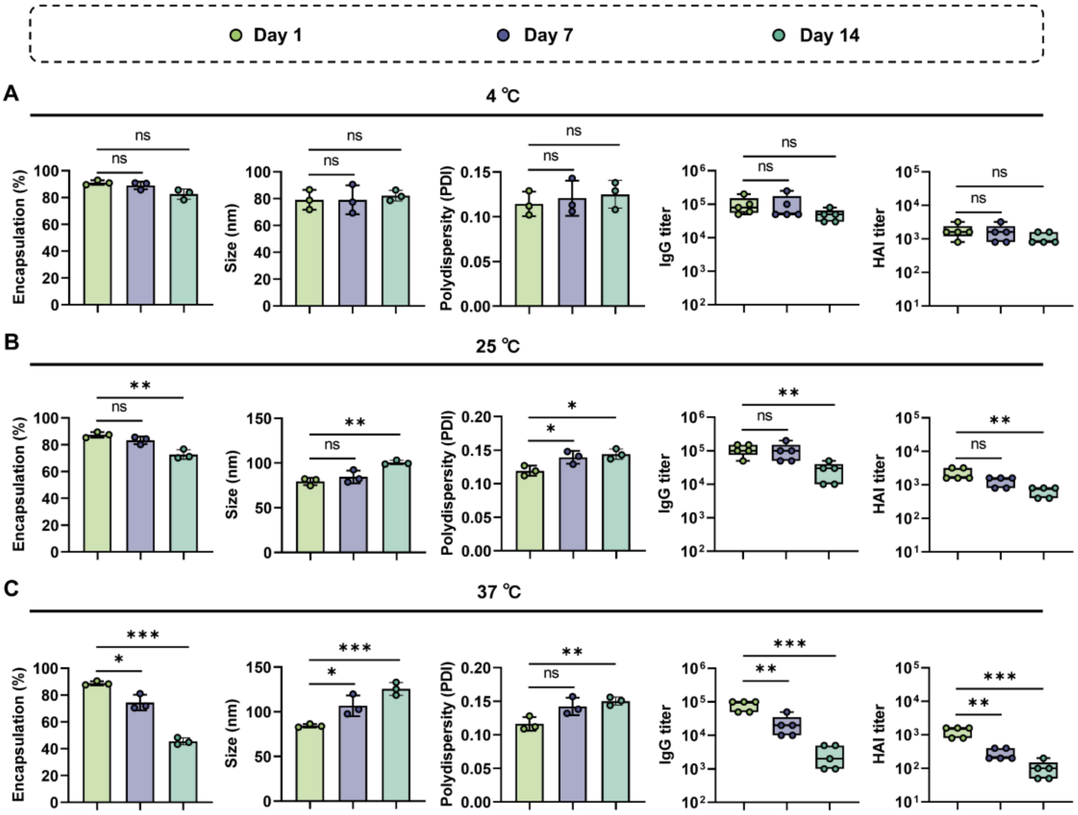

Fig. S12. Thermostability of HA-CXCL13-circRNA tLNPs formulations under different temperatures. (A-C) HA-CXCL13-circRNA tLNPs was stored at 4 °C (A), 25 °C (B), or 37 °C (C) for 1, 4, and 7 days, and the encapsulation efficiency, particle size, and PDI were assessed. BALB/c mice were intramuscularly vaccinated on day 0 and day 14, and IgG and HAI titers were measured at week 5 post-primary immunization. Data are represented as the mean ± S.D. Unpaired two-tailed Student's *t* test was performed for comparison, as indicated in the figures; *$p < 0.05$; **$p < 0.01$; ***$p < 0.001$; ns, not significant.

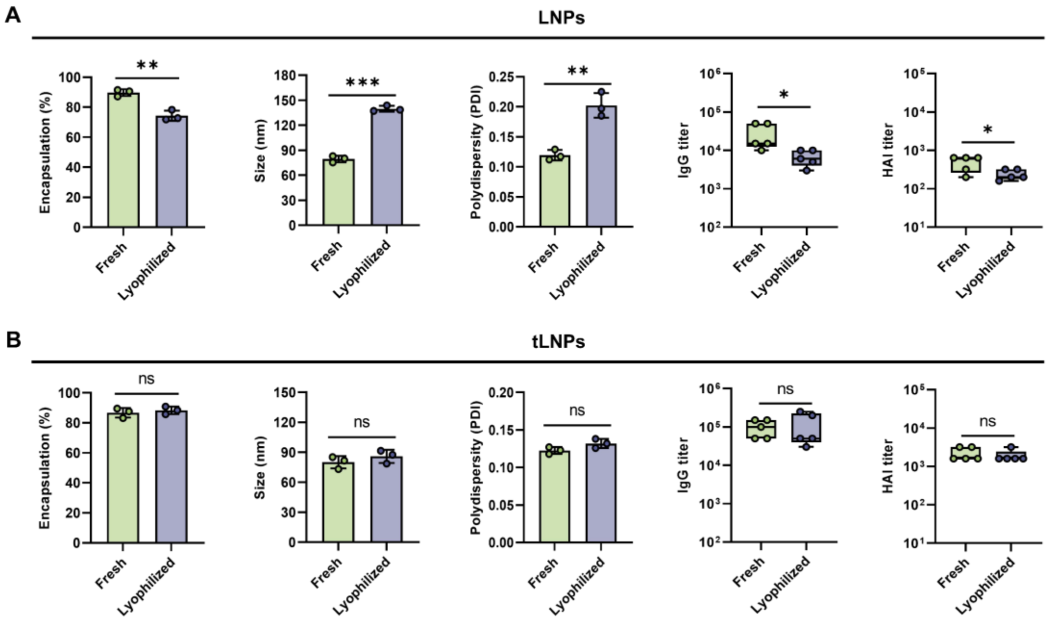

Fig. S13. Antibody modification improved the stability of LNPs after lyophilization. The lyophilized HA-CXCL13-circRNA LNPs (A) and HA-CXCL13-circRNA tLNPs (B) were rehydrated for analysis of their particle size, encapsulation efficiency, and PDI. Additionally, mice were immunized on both day 0 and day 14, and serum samples were collected at week 5 post-primary immunization for antibody detection. Samples were supplemented with 8% sucrose as a cryoprotectant prior to lyophilization. Data are represented as the mean ± S.D. Unpaired two-tailed Student's *t* test was performed for comparison, as indicated in the figures; $*p < 0.05$; $**p < 0.01$; $***p < 0.001$; ns, not significant.

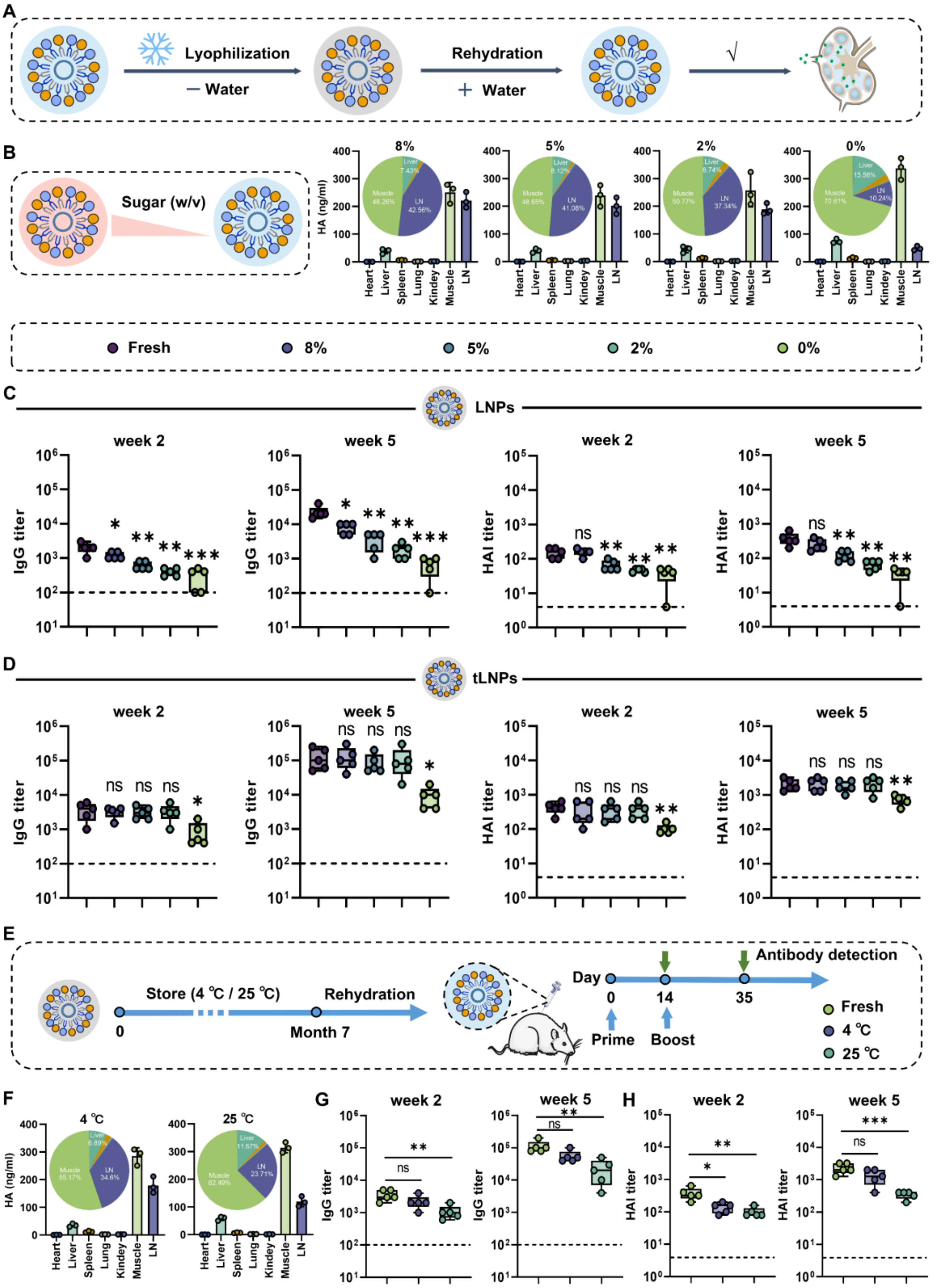

Fig. S14. Antibody modification imparts lyophilization stability and targeted stability to the LNPs. (A) Schematic diagram of lyophilization and rehydration of the targeted-LNPs (tLNPs) vaccine. (B) HA distribution of HA-CXCL13-circRNA tLNPs formulations containing different sucrose concentrations after lyophilization. (C-D) Immunogenicity of HA-CXCL13-circRNA LNPs and HA-CXCL13-circRNA tLNPs formulations containing varying sucrose concentrations after lyophilization. (E-H) Long-term storage stability evaluation. The HA-CXCL13-circRNA

tLNPs formulations containing a 2% sucrose concentration was lyophilized and stored for 7 months at either 4 °C or 25 °C conditions, followed by immunogenicity assessment (E). Biological distribution of the antigen in different tissues (F). Antibody level (G-H). N = 5 mice per group. Data are represented as the mean ± S.D. Unpaired two-tailed Student's *t* test was performed for comparison, as indicated in the figures; **p* < 0.05; ***p* < 0.01; ****p* < 0.001; ns, not significant.

SI References

1. L. Qu *et al.*, Circular RNA vaccines against SARS-CoV-2 and emerging variants. *Cell*. **185**, 1728-1744 (2022).
2. N. Dammes *et al.*, Conformation-sensitive targeting of lipid nanoparticles for RNA therapeutics. *Nat Nanotechnol*. **16**, 1030-1038 (2021).
3. N. N. Zhang *et al.*, A Thermostable mRNA Vaccine against COVID-19. *Cell*. **182**, 1271-1283 (2020).
4. F. Borriello *et al.*, An adjuvant strategy enabled by modulation of the physical properties of microbial ligands expands antigen immunogenicity. *Cell*. **185**, 614-629 (2022).
